# A grey-box modelling methodology to express home heat-energy usage as statistical distributions — case studies in urban Ireland

**DOI:** 10.1007/s12053-022-10038-9

**Published:** 2022-05-31

**Authors:** Paul Beagon, Fiona Boland

**Affiliations:** 1 Sustainable Energy Authority of Ireland, 3 Park Place, Hatch Street Upper, Dublin 2, Ireland; 2 Data Science Centre, Division of Biostatistics and Population Health Sciences, Royal College of Surgeons in Ireland, 123 St Stephen’s Green, Dublin 2, Ireland

**Keywords:** Home energy retrofit, Building performance simulation, Modelica, AixLib library, Weibull distribution, Normal distribution, Distribution goodness-of-fit, Anderson–Darling test

## Abstract

Home energy retrofit has recurred in public policy throughout recent decades. However, the savings in energy usage attributable to home retrofit have remained difficult to accurately predict. Occupants cause prediction inaccuracies by varying different factors, especially heating setpoints temperatures and heating patterns. Acting together, such occupant factors result in distributions — not single values — of heat-energy usage, even among similar homes. Datasets of heat-energy distributions can be found by building performance simulation using modern grey-box models. This study presents a methodology to simulate grey-box models of home heating through ranges of heating setpoints and patterns. An entire process to calibrate, validate and simulate at a large scale is described, and then demonstrated using case studies. Grey-box models, written in Modelica language, can conveniently simulate through large ranges of occupant factors. The case studies exploited this advantage of grey-box models to simulate empirical data on occupant factors. (For instance, empirical data found that home heating setpoints shifted before and after home energy retrofit.) In doing so, the datasets of simulation results enabled the exploration of home heat-energy usage with the normal and Weibull statistical distributions. Additionally, the heat-energy distributions of case-study homes were statistically tested, first for retrofit savings, second for equality to each other and third for equality to an official heat-energy estimate. Results demonstrate that home heat-energy usage, at a large scale, is best expressed as a Weibull distribution not normality. After home energy retrofit, heat-energy usage displays less variation (in general), less skewness, and thus becomes closer to normality. Occupant factors were found to vary home heat-energy usage into distinct distributions, even within similar homes. Therefore, in most case-study homes, heat-energy usage did not equal an official estimate. Finally, shallow retrofit of a modern home in Ireland fails to save heat-energy usage by most occupants.

**Supplementary Information:**

The online version contains supplementary material available at 10.1007/s12053-022-10038-9.

## Introduction

A consensus has emerged on the need for home energy retrofit (HER) and its delivery at large scale (Gupta et al., 2015; Mathiesen et al., 2016; McKenna et al., 2013). Proponents of HER foresee its contribution to different goals: reducing greenhouse gas, combatting energy poverty, — in addition to —, saving energy (DCCAE, 2019; Dixon & Eames, 2013; Fylan et al., 2016; Sdei et al., 2015). Despite these august goals, a gap remains in predicting the energy-usage savings attributable to HER. One prediction approach is to model homes before and after retrofit, and then simulate their performance (Swan & Ugursal, 2009).

Building performance simulation (BPS) can predict home energy usage under different scenarios, but often fail to incorporate heating variations caused by occupants (Huebner et al., 2013; Shipworth et al., 2010). Inaccurate model parameters, such as air changes per hour (ACH) or building fabric properties, further compound the inaccuracies in simulated energy usage. Occupants cause the gap between simulated and measured energy usage for two main reasons during simulation: (1) unrealistic internal temperatures (Jones et al., 2016; Teli et al., 2016) and (2) failure to apply dominant occupancy profiles (Zahiri & Elsharkawy, 2018). Put simply, different occupants operate homes differently.

Occupants also affect HER by causing the *rebound effect*. In the context of home heating, rebound effect is post-retrofit overheating or “temperature take back” (Sorrell et al., 2009), also known as “comfort taking” (Hamilton et al., 2016). Temperature take back occurs when occupants prefer warmer internal temperatures instead of maximising energy and financial savings. In extreme cases, the rebound effect may negate energy efficiency savings, an outcome termed “backfire” where the intervention actually increases overall energy usage (Druckman et al., 2011). Druckman et al. found that the rebound effect results in only a portion of the greenhouse gas (GHG) reductions estimated by engineering calculations. Furthermore, the largest *percentage* rebound hampered potential GHG reductions by low-income households, according to UK analysis (Chitnis et al., 2014). That analysis found that the low-income households were mainly affected by *direct* rebound, whereby occupants use more of an energy service after an intervention decreases its effective price.

In summary, the differences in home operation mean that a single energy-usage value cannot represent every home, even structurally similar homes comprising a neighbourhood. A decade ago, neighbourhoods were proposed as a spatial scale offering the highest potential for large-scale HER (Koch & Girard, 2011; Koch et al., 2012). Koch & Girard defined neighbourhoods as groups of structurally similar homes — proposing that their energy usage is best expressed as *statistical distributions*. This paper now presents a methodology and case studies to calculate the heat-energy usage by structurally similar homes as a statistical distribution.

### Statistical distributions of home energy usage

Previous research has expressed home energy usage as statistical distributions (Irwin et al., 1986; Munkhammar et al., 2014). Both research papers describe how meter data was used to find distributions of electricity usage — an energy *vector*. Energy vectors allow transfer, in space and time, a given quantity of energy, hence making it available for use distantly (Orecchini & Santiangeli, 2011). When the energy quantity is used, it provides useful energy services such as heating, cooling, transport and so on. In contrast to the previous research, this study finds the statistical distribution of heat-energy usage for space heating — an individual energy *service*. Furthermore, this study compares heat-energy distributions before and after HER. In contrast, savings in electricity usage are difficult to achieve by HER because of demand from multiple energy services, such as lighting, cooking and supplementary heating (Irwin et al., 1986), and nowadays dishwashers and other appliances (Munkhammar et al., 2014).

**Fig. 1:**
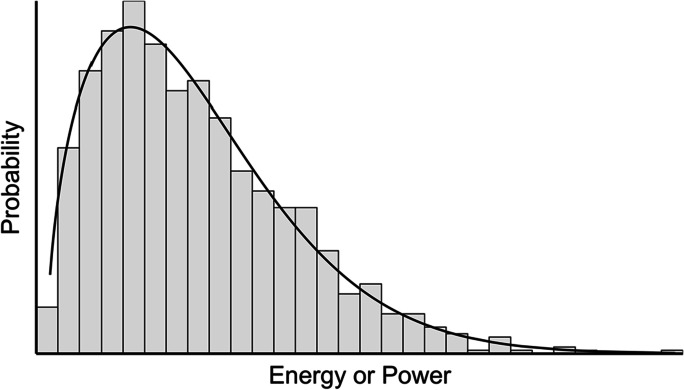
Positively skewed histogram and fitted Weibull distribution

Both Irwin et al. and Munkhammar et al. fitted Weibull distributions to positively skewed datasets of electricity usage. Unlike the popular normal distribution, a Weibull distribution covers only positive values in different asymmetric shapes. Figure 1 displays a Weibull distribution fitted to a positively skewed histogram.

Multiple techniques exist to fit a distribution to a dataset of energy values. Munkhammar et al. used the Kolmogorov-Smirov (KS) test to check the fit of electricity usage to Weibull and log-normal distributions. A similar test by Anderson–Darling (AD) improves the KS test by weighting the values at the distribution tails (Keller, 2011), where non-normality often manifests (Nelson, 2018). Therefore, the AD test is capable of detecting the differences between overlapping normal and Weibull distributions (Engmann & Cousineau, 2011). For these reasons, this study used the AD test for distribution fit. Abbreviations, including KS, AD and others, plus symbols are organised in Table 1.

**Table 1:** Abbreviations and symbols

**Abbreviations**	
ACH	Air changes per hour
AD	Anderson–Darling, a goodness-of-fit test for statistical distributions
ASHRAE	American Society of Heating, Refrigeration and Air-Conditioning Engineers
BPS	Building performance simulation
CI	Confidence interval, 95% used in this study
CSO	Central Statistics Office
CV(RMSE)	Coefficient of variation of the root mean square error
DASSL	Differential / algebraic system resolver
DEAP	Dwelling Energy Assessment Procedure
EU	European Union
GHG	Greenhouse gas
GOF	Goodness-of-fit
HER	Home energy retrofit
IWEC	International Weather for Energy Calculation
KS	Kolmogorov-Smirov, a goodness-of-fit test for statistical distributions
kWh	kilowatt hour
MFH	Multi-family house
NMBE	Normalised mean bias error
NREL	National Renewable Energy Laboratory
PID	Proportional-integral-derivative
PDF	Probability distribution function
QQ	Quantile–quantile
RC	Resistance–capacitance
SFH	Single-family house
SEAI	Sustainable Energy Authority of Ireland
Std dev	Standard deviation
TABULA	Typology Approach for Building Stock Energy Assessment
**Symbols**	
${\alpha }_{conv}$	Convective heat transfer coefficient
${\alpha }_{rad}$	Radiative heat transfer coefficient
h	Hour
m	Mean of measured data
m_i_	Measured data, indexed by i
m_r_	Statistical moment of r^th^ order
n	Sample size
p	Number of parameters
s	Standard deviation of a sample
s_i_	Simulated data, indexed by i
$\overline{x }$	Mean average of a dataset x, for example energy use
$\gamma$	Skewness of a statistical distribution

### Grey-box models for buildings

Building models can be categorised into three general approaches: white-box, black-box and grey-box. The grey-box approach combines the physics of the white-box approach, and the statistics or machine learning of the black-box approach. Functional labels for the three approaches are “physical”, “machine learning” and “hybrid” respectively (Foucquier et al., 2013). Two decades have passed since a grey-box model was used to represent building envelopes (Déqué et al., 2000). In the meantime, other studies have quantified uncertain parameters in building models using historical time-series data (Bacher & Madsen, 2011; Brastein et al., 2018; Harb et al., 2016; Reynders et al., 2014). This process is known as parameter estimation or “tuning” and is used to calibrate grey-box models.

One type of grey-box building models, called lumped-capacitance, has received much attention and been evaluated (Vivian et al., 2017). That evaluation described how lumped capacitance models simplify (or lump) a building’s distributed thermal mass into a discrete number of thermal capacitances. Within each model, thermal resistances interconnect the thermal capacitances forming thermal networks; hence, lumped-capacitance is often referred to as “thermal networks”, “resistance–capacitance” or simply “RC”. Henceforth, lumped-capacitance or thermal network models are referred to as “RC models”. RC models are considered grey-box if their parameters are estimated or “tuned” using available weather and energy-use datasets, as opposed to analytical calculation. This study incorporated parameter estimation into its Methodology (section Calibration procedure and validation).

After model calibration, the system domain of RC models “can be solved analytically, thus avoiding problems of convergence and stability” (Vivian et al., 2017). Vivian et al. concluded that RC models “reliably calculate” overall energy demand but only high-order models, defined by many thermal capacitances, can calculate transient hourly demands. Low-order models are, however, easier to solve during simulation. Easier simulation enables scaling up building simulation to city district (Lauster et al., 2014a, b), or to urban energy in an arid climate (Zekar & El Khatib, 2018). The latter research found that RC models of cooling load provided a “limited loss of accuracy” and “satisfying” level of performance. Nowadays, grey-box models still offer the three main advantages identified two decades ago (Déqué et al., 2000), with minor updates:

- Limited number of parameters to be found prior to simulation,
- Fewer equations to solve during simulation, enabling larger spatial scale and
- Flexibility to detail a model subsystem amongst other model components.

### Modelling home archetypes to calculate energy usage

Having introduced grey-box building models, definition of the homes represented by the models is necessary. For this study, separate building models must represent the same home in two states: before and after home energy retrofit. Henceforth referred to as *as-built* and *retrofit* states. As already mentioned, homes in the same neighbourhood are structurally similar. Thus, one building archetype can define the structure and fabric of many similar homes forming a neighbourhood.

The archetype approach has been used to understand the *aggregated* impact of energy efficiency policies and technologies (Monteiro et al., 2017; Reinhart & Cerezo Davila, 2016). Similar to this study, Monteiro et al. selected the neighbourhood spatial scale, delineating four neighbourhoods by construction period and major urban intervention. They also identified three stages in archetype generation: *classification*, *parameterisation*, and *modelling*. Regarding the first stage of archetype generation, an existing classification of European homes is now introduced.

### Archetypes from TABULA typology

Project TABULA, conducted during 2009–2012, was formally named “Typology Approach for Building Stock Energy Assessment”. It was the second of three Intelligent Energy Europe projects, including DATAMINE (2006–2008) and EPISCOPE (2013–2016). A project summary (Loga, 2010), answers the question “What is TABULA about?” as follows:

*The objective … is to create a harmonised structure for European Building Typologies. Each participating country will on that basis develop a National Building Typology, that is a set of model buildings with characteristic energy related properties. The project focuses on residential buildings …*

**Fig. 2:**
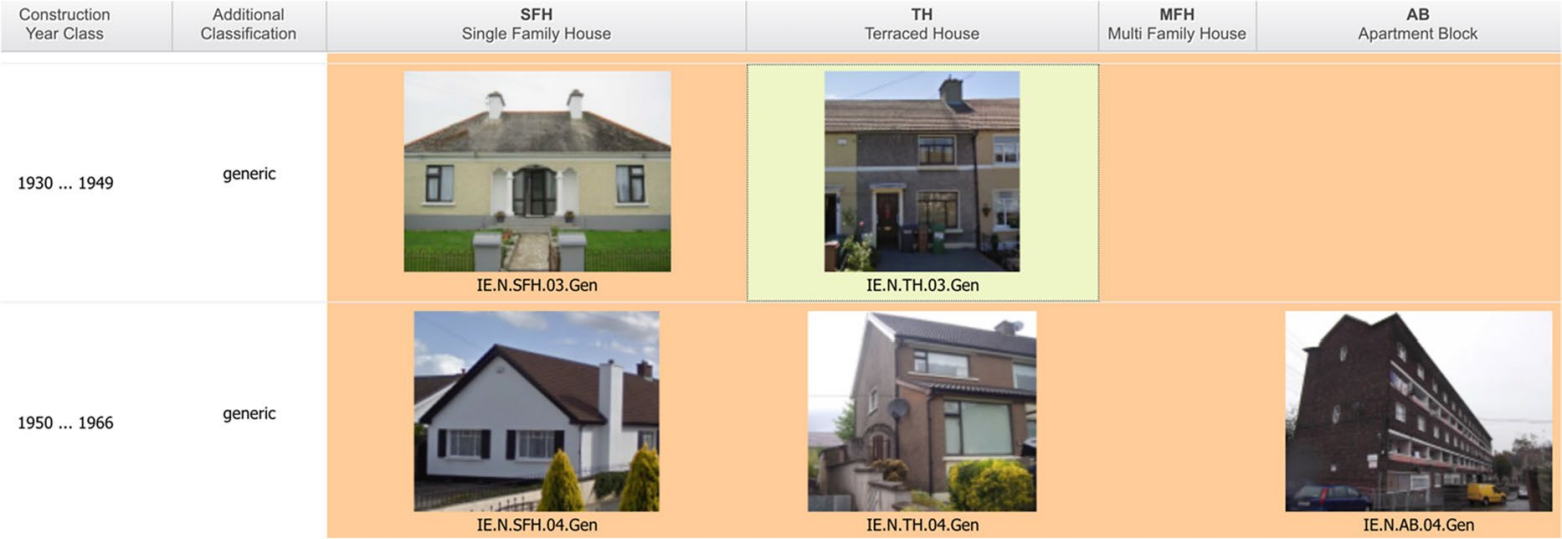
TABULA webtool displaying an extract of Ireland’s building matrix. The highlighted cell contains a single-family house archetype TH.03.Gen: a terraced house constructed during 1930–1949 (TABULA, 2016)

Each National Building Typology produced by TABULA is a “Building Type Matrix” hereinafter “building matrix”, that standardised the classification of residential buildings (Loga et al., 2016). This study used Ireland’s building matrix in section Home archetypes of the Methodology. Figure 2 displays the standardised matrix axes:

- Building age classes: the construction period of the building.
- Building size classes: clustering the buildings by size, such as single-family houses.

Each home archetype within the building matrix was accompanied by a complete dataset comprising general features such as number of storeys, geometric data of volume and envelope area, thermal properties such as U-values and supply system features such as heat generator efficiency (Loga, 2010). Supply systems pertaining to home energy retrofit were the home heating and cooling systems. TABULA classified heating and cooling systems into three types: (1) initial reference system, termed “as-built” in this study, (2) standard retrofit or (3) advanced retrofit. TABULA archetypes also included energy-use parameters such as air change rates and heating fuels. Simple occupant schedules were assumed by TABULA, thus *not used* in this study because they lacked the variation caused by occupants. TABULA assumed a single Irish climate zone but allocates multiple climate zones to larger countries, for instance three zones to Italy.

By focusing on homes, TABULA attempted to predict the impact of energy efficiency measures and select effective strategies for home energy retrofit (Ballarini et al., 2014; Loga et al., 2016). Estimates of building energy relied on a quasi-steady-state calculation over an entire season (TABULA, 2013). Both archetype definitions and energy calculations were published online (TABULA, 2016), and were adopted by other research into Belgium homes (Reynders et al., 2014) and six other countries (Coma et al., 2019).

### Home types in Ireland

The home archetypes defined in TABULA reflect the popularity of single-family houses (SFHs) in Ireland, over multi-family houses (MFHs). Furthermore, Ireland’s 2016 census confirmed the popularity of SFHs and disaggregated their quantities by type: detached, semi-detached and terraced (CSO, 2020).

**Fig. 3:**
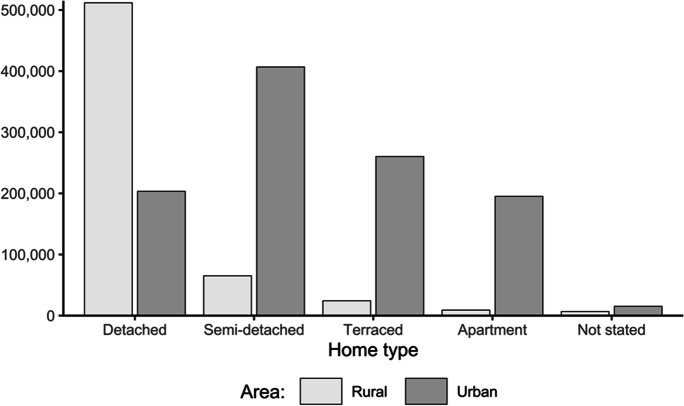
Numbers of Irish rural and urban home types in 2016. Single-family houses exceed 80% of urban homes

Disaggregated numbers of home types in Table 2 reveal that detached houses comprised over 80% of *rural* households, motivating previous research into their retrofit (Ahern et al., 2013). In contrast, the 2016 census reported that *urban* or “town” households span the three SFH types more evenly, with semi-detached and terraced houses dominating (Fig. 3). The numbers of urban homes in descending order of house type were 406,798 semi-detached, 260,319 terraced and 203,346 detached (CSO, 2020). Given that large-scale retrofit applies to urban areas, this study’s case studies focus on the popular semi-detached and terraced home types.

**Table 2:** Home-type numbers in Ireland, split by rural and urban areas (CSO, 2020). Bed-sit numbers are combined into apartment numbers

Home type	Rural area (number)	Urban area (number)	Total (number)
Detached	511,787	203,346	715,133
Semi-detached	65,150	406,798	471,948
Terraced	24,250	260,319	284,569
Apartment	8997	195,148	204,145
Not stated	6644	15,226	21,870
Total	616,828	1,080,837	1,697,665

Apartments or other multi-family homes (including bed-sits) constituted 195,148 (under 20%) of all urban households, leaving 15,226 home types not stated (CSO, 2020). Another factor in home energy usage is that Census 2016 recorded an increasing number of persons per household, reversing the downward trend since 1966 (CSO, 2018). Moreover, increasing household sizes were confined to urban areas.

Turning to the causes of dominant home types, the construction of single-family houses permeated both rural and urban areas of Ireland during the last century. McManus (2011) cited “The typical Irish home, whether in the city or countryside, is a one or two storey, single or semi-detached cottage…” (Pfretzschner, 1965); before remarking that Pfretzschner’s insight described Irish housing in *any* twentieth century decade as follows:

*The predominance of the standardised three- or four-bedroom, semi-detached or detached house, was not challenged until the 1990s when there was a surge in apartment provision.*

This characterisation of semi-detached and detached homes as “standardised” implies their structural similarity and suitability for classification into home archetypes. Multi-family homes, such as apartments, remained a minor part of Irish twentieth century housing supply. Apartments were perceived as working class, associated with overcrowded tenements, and less popular than houses (McManus, 2011). An official recommendation for the aforementioned “cottages”, i.e. suburban two-storey houses, instead of urban flats appeared in the 1939/43 Report of Inquiry (Rowley, 2015). Thus, purpose-built apartments gradually reached 5% of new builds during the 1980s, only ramping up construction rates during the 1990s (Energy Action, 2014). The resultant 85% growth in apartments during 2002–2016, captured in the census (CSO, 2018), means the average apartment is significantly more modern than the average single-family house.

In addition to older construction age, single-family houses dominate heat-energy usage due to their larger average size and higher specific heat demand. A review of building stock retrofit in Germany forecasted that single-family houses will account for approximately two thirds of heat demand until 2050 (McKenna et al., 2013). Hence, this study selects single-family homes constructed before the 1997–2002 changes in building regulations (Dineen & Ó Gallachóir, 2011), as those most likely to benefit from home energy retrofit. However, the benefits attributable to HER vary because of the variation in energy usage caused by occupants. For instance, different occupants prefer different internal temperatures controlled by *setpoints,* and warm their homes at different times of the day called *heating patterns*. Since setpoints and heating patterns are critical factors in home heating calculations, they were empirically defined in two Methodology sections: Data sources for simulations, and Heating and internal gains schedules.

### Calculation of energy savings attributable to HER

As already mentioned, TABULA defined standard *and* advanced retrofits per archetype that differed in their upgrade of each archetype’s heat generator (TABULA, 2016). A standard retrofit upgraded a boiler, whereas an advanced retrofit replaced a boiler *and* switched fuel by deployment of an electrical heat pump. To remain independent of fuel type, energy savings attributable to retrofit were measured in sensible heat energy for space heating. This study analyses the *sensible heat energy* used to provide one energy service: *space heating*, before and after a *deep retrofit*. The three preceding terms are now defined:

*Sensible heat energy* or *heat energy* flows as convective energy from heating equipment to maintain internal air at a heating setpoint. For brevity, *sensible heat energy* is hereinafter shortened to *heat energy*.

*Space heating* is the heat energy required to offset the thermal losses across the building envelope by conduction and radiation, as well as air infiltration and ventilation, in an effort to maintain the living space at a comfortable temperature (Swan & Ugursal, 2009).

*Deep retrofit* combines multiple measures to improve the building envelope fabric and upgrade heat generation. Once a building envelope is made consistent, it reduces outside air infiltration and other thermal losses that must be offset by space heating. In contrast, *shallow retrofit* comprises one or two maintenance measures, such as increased boiler efficiency or new glazing. Deep-retrofit designs are often implemented to deliver policy-driven savings in energy or GHG emissions. Policy-driven targets vary around 60% savings (Arcipowska et al., 2017), but have extended to 75% savings (GBPN, 2013).

### Contribution

This study contributes to research by providing a methodology and case study examples to parameterise and evaluate the statistical distributions of heat-energy usage for home space heating. Such distributions of heat-energy usage are particularly relevant to large-scale home energy retrofit, for example at neighbourhood scale. While the case studies are in Ireland, the methodology can be applied internationally, most straightforwardly in countries covered by the TABULA research project. The preceding Introduction covered the points supporting the contribution: the need for home energy retrofit and its large-scale delivery.

In addition, this study contributes to the *research gap* that building energy models typically lack empirical data on internal temperature and heating duration. Furthermore, the same models ignore occupant self-rationing known as the prebound effect (Sunikka-Blank & Galvin, 2012). These findings apply to homes in general (Huebner et al., 2013; Shipworth et al., 2010), and social housing in particular (Jones et al., 2016; Teli et al., 2016; Zahiri & Elsharkawy, 2018). Therefore, empirically sourced heating temperature setpoints and heating patterns are defined in two sections: Data sources for simulations and Heating and internal gains schedules. In total, the aims of this study are fourfold:

1. Present a calibration and validation methodology for grey-box models coded in Modelica language, using heat-energy usage data from simulation of equivalent white-box models.
2. Convert the heat-energy usage from the simulations into statistical distributions and test their fit to the normal and Weibull distributions.
3. Explore the different heat-energy distributions, resulting from different heating setpoints and patterns operated by occupants.
4. Use the distributions to calculate mean confidence intervals (CIs) and test the hypothesis that heat-energy usage actually equals an official single value.

The following Methodology covers the selection, calibration, and large-scale simulation of grey-box models that represent home heating. To simulate different occupant operation, different heating setpoints and heating patterns are defined. Both definitions originate from published empirical data. The resulting distributions and statistics of heat-energy usage by the modelled homes are presented and explored in the Results and Discussion. Finally, the paper closes with Conclusions that include implications and future work.

## Methodology

**Fig. 4:**
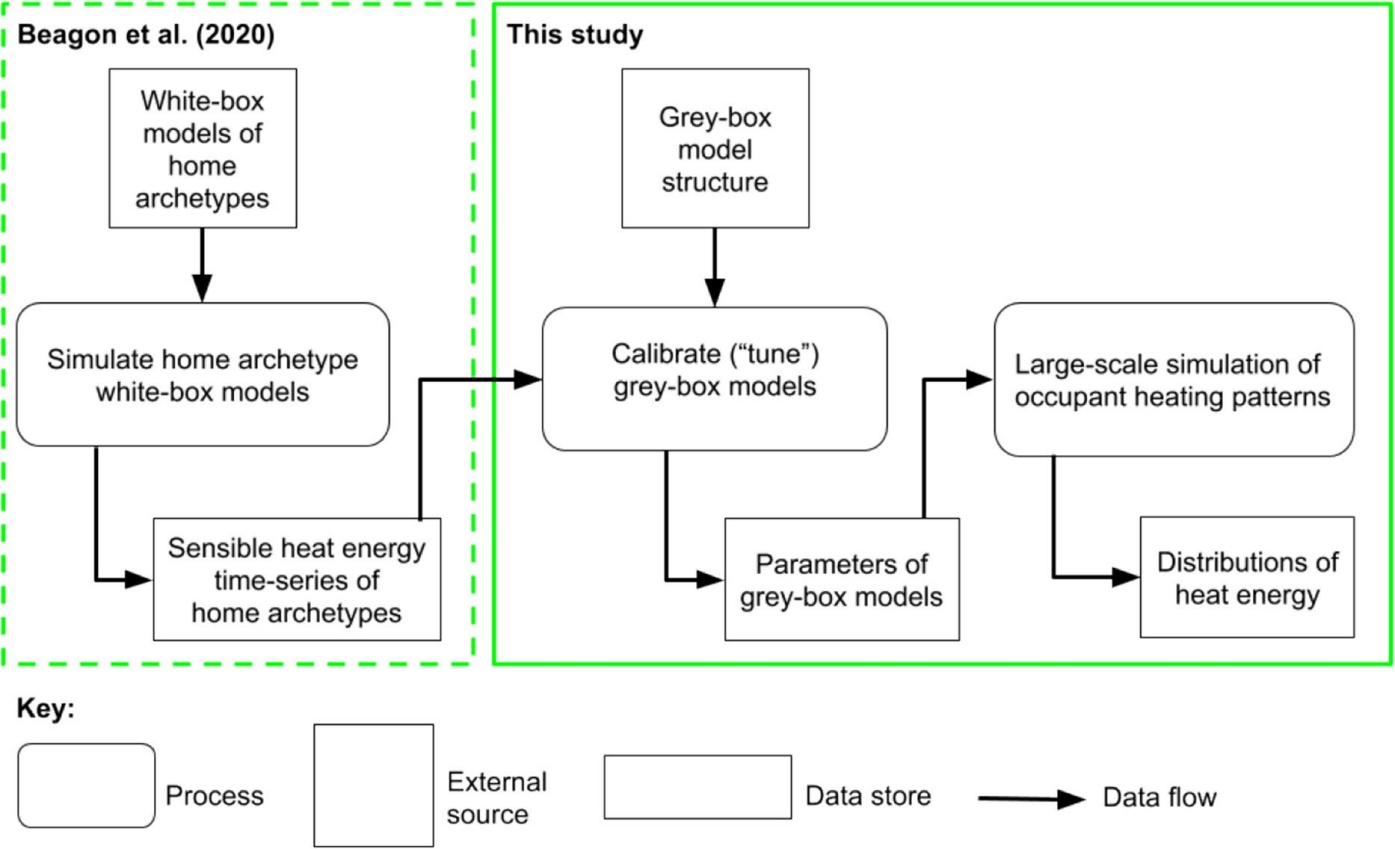
Data flow diagram of modelling and simulation by this study

After the structure of a grey-box model was selected, it was calibrated for each home archetype. Calibration of grey-box models relied on the energy usage simulated by white-box models of the same home archetypes. Both white-box and grey-box models were simulated with identical weather files, internal gains, ventilation and infiltration; for more details, see the companion paper (Beagon et al., 2020). That paper details the creation of the white-box models — the first step in the methodology flow undertaken by this study (Fig. 4).

### Home archetypes

Since this study investigated large-scale HER, for example at neighbourhood scale, the methodology selected *urban* home archetypes. As discussed already in section Home types in Ireland, single-family homes dominate Irish housing, primarily as semi-detached and terraced houses in urban areas.

TABULA’s typography classified both semi-detached and terraced homes as “terraced”; identified by the letters “TH” within the archetype names (TABULA, 2016). The three archetypes selected as case studies are hereinafter referred to as “TH03”, “TH06” and “TH07”. The archetypes differed by their construction periods, where TH03 and TH07 are the oldest and youngest respectively (Table 3). All three archetypes shared the same urban area of Dublin, Ireland’s capital city. According to the home archetype brochure, TH03 was “very common” in Dublin’s 1930s and 1940s building stock, TH06 was “commonly built” in Dublin during 1978–1982 and TH07 was a “very typical house” built in Dublin during the 1980s (Badurek et al., 2014).

**Table 3:** Summary details of case-study home archetypes, including unique construction year class and building type of either terraced or semi-detached

Archetype	Full name	Construction year class	Building type	Additional classification
TH03	IE.N.TH.03.Gen	1930–1949	Terraced	Mass concrete
TH06	IE.N.TH.06.HBlock	1978–1982	Semi-detached	Hollow block
TH07	IE.N.TH.07.Hblock	1983–1993	Terraced	Hollow block

### Selection of Grey-box model

Section Grey-box models for buildings introduced thermal or RC networks to model building performance. Each grey-box model in this study represented the space heating of a home archetype as one system, containing a RC network to simplify the building fabric subsystem. By simplifying the building fabrics, the grey-box models facilitated the many simulations across a range of heating setpoints and heating patterns. As expected, the range of setpoints and patterns resulted in statistical distributions of heat-energy usage — the main results of this study.

All grey-box models were implemented in Modelica code and simulated in the Dymola environment using its default DASSL solver (differential algebraic system resolver). This study used the Modelica library *AixLib* and its subsystems (Müller et al., 2016) to implement an existing RC network structure (Lauster et al., 2014a, b). That model separated equivalent air temperatures of wall and windows for each building orientation. Thereby, the effect of solar radiation was more accurately calculated, including long-wave radiation (Lauster et al., 2014a).

Radiative heat traversed the different components of the building fabric. For instance, heat energy transferring from windows to internal walls was impeded by resistor R_WinIntRad_. Specific resistor values are based on a radiative heat transfer coefficient (${\alpha }_{\mathrm{rad}}$), assumed constant in the simulated temperature range at 5 W/(m^2^K). Concerning convective heat transfer, a coefficient (${\alpha }_{\mathrm{conv}}$) of 2.7 W/(m^2^K) is assumed. Both ${\alpha }_{\mathrm{rad}}$ and ${\alpha }_{\mathrm{conv}}$ values were sourced from (Lauster et al., 2014a, b). The ${\alpha }_{\mathrm{conv}}$ coefficient determines resistances, for example, convective heat transfer at windows R_WinCon_. Other resistances and capacitances were specific to the house fabric construction: external wall (ext), internal walls and objects (int), and floor plate (floor).

**Fig. 5:**
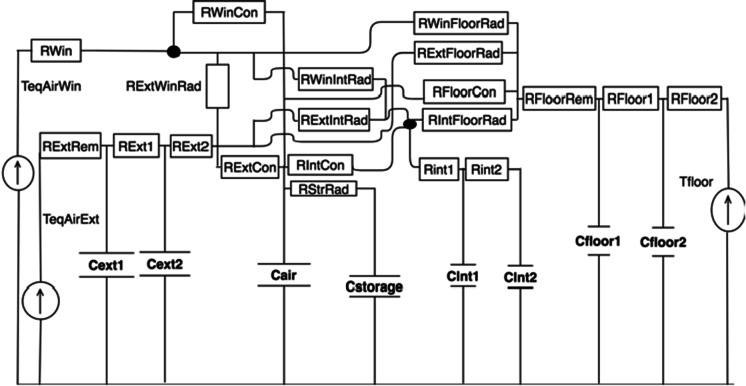
Thermal network (RC) subsystem representing the building fabric of a house, adapted from (Lauster et al., 2014a, b). Walls and floors are configured as 3R-2C components

In order to reduce the over-responsiveness of simulated internal temperatures, lumped capacitances were split in two (Tindale, 1993). For example, the total external wall capacitance was apportioned between C_ext1_ and C_ext2_ that interconnected by resistors forming a 3R-2C component. These external wall components were ordered as: R_extRem_, C_ext1_, R_ext1_, C_ext2_, R_ext2_ (Fig. 5). The resistance (and some capacitance) values of the external wall increased after retrofit. Such increases reflect the fabric retrofit measures of wall insulation and glazing replacement. The next section, Automated model calibration, describes how this study quantified these RC values by “tuning” grey-box models to datasets of white-box models’ heat-energy usage.

### Automated model calibration

Model calibration is the process of “tuning” selected model parameters to obtain a better agreement between the model’s simulated or *predicted* behaviour and observed or *measured* behaviour (Dassault Systèmes AB, 2017). In this study, selected parameters of the grey-box models were tuned to match the simulated heat-energy usage by the white-box models.

A *calibrate* task resides in the Dymola optimisation library for Modelica (Elmqvist et al., 2005). This task iterated through the simulation of a grey-box model and compared its energy-usage dataset to those from previous simulations of white-box models (Beagon et al., 2020). By iterating through simulations and comparisons, an optimisation was achieved by automatic re-estimations of selected model parameters. The optimisation objective was to achieve minimum error between the two datasets of heat-energy usage. The selected parameters for tuning were the three external wall resistors and its two capacitances: R_ext1_, R_ext2_, R_extRem_, C_ext1_ and C_ext2_ (Fig. 5). After updating the tuned parameters with their new estimated values, the grey-box models were deemed calibrated.

### Model validation indices

Validation of calibrated models used two goodness-of-fit (GOF) indices. The errors between *monthly* heat-energy usage from simulations of white-box and calibrated grey-box models were quantified by both indices. First, the coefficient of variation of the root mean square error (CV(RMSE)) quantified the variation of errors. Second, normalised mean bias error (NMBE) quantified the average of errors. In other words, CV(RMSE) and NMBE quantify the standard deviation and mean of the errors between two datasets (Reddy & Maor, 2006).

Referring to Eqs. (1) and (2), $\overline{m }$ represents the mean of measured data. Throughout this study, the existing white-box dataset were represented by the “measured” data of month i (m_i_), whereas the grey-box model produced the “simulated” data of month i (s_i_). Since both models produced monthly results, variable n equalled twelve. Finally, *p* was the number of adjustable model parameters (Robson & McCartan, 2016); however, this study adopted proposed values: *p* = 1 for CV(RMSE) and *p* = 0 for NMBE (Reddy & Maor, 2006).

1 $$\mathrm{CV}\left(\mathrm{RMSE}\right) = \frac{1}{\overline{m} }\sqrt{\frac{\sum_{i=i}^{n}{\left({m}_{i}-{s}_{i}\right)}^{2}}{n-p}}$$

2 $$\mathrm{NMBE} = \frac{1}{\overline{m} }\sqrt{\frac{\sum_{i=i}^{n}\left({m}_{i}-{s}_{i}\right)}{n-p}}$$

ASHRAE^1^ Guideline 14 stipulates two necessary GOF validation criteria: CV(RMSE) < 15%, *and* -5% < NMBE < 5% (Reddy & Maor, 2006). The larger CV(RMSE) criterion reflects its absolute measurement of all errors (1), whereas the smaller NMBE criterion reflects its cancelling out of positive and negative bias (2).

### Calibration procedure and validation

Validated grey-box models met the ASHRAE validation criteria using *monthly* heat-energy usage results from two simulations. One simulation was under a static “steady-state” schedule, whereas the second simulation was under the dynamic DEAP (Dwelling Energy Assessment Procedure) schedule. The steady-state simulations had constant heating, but an unoccupied home. The DEAP simulations had heating and an occupied home during the morning and evening only (07:00–09:00 and 17:00–23:00).

**Fig. 6:**
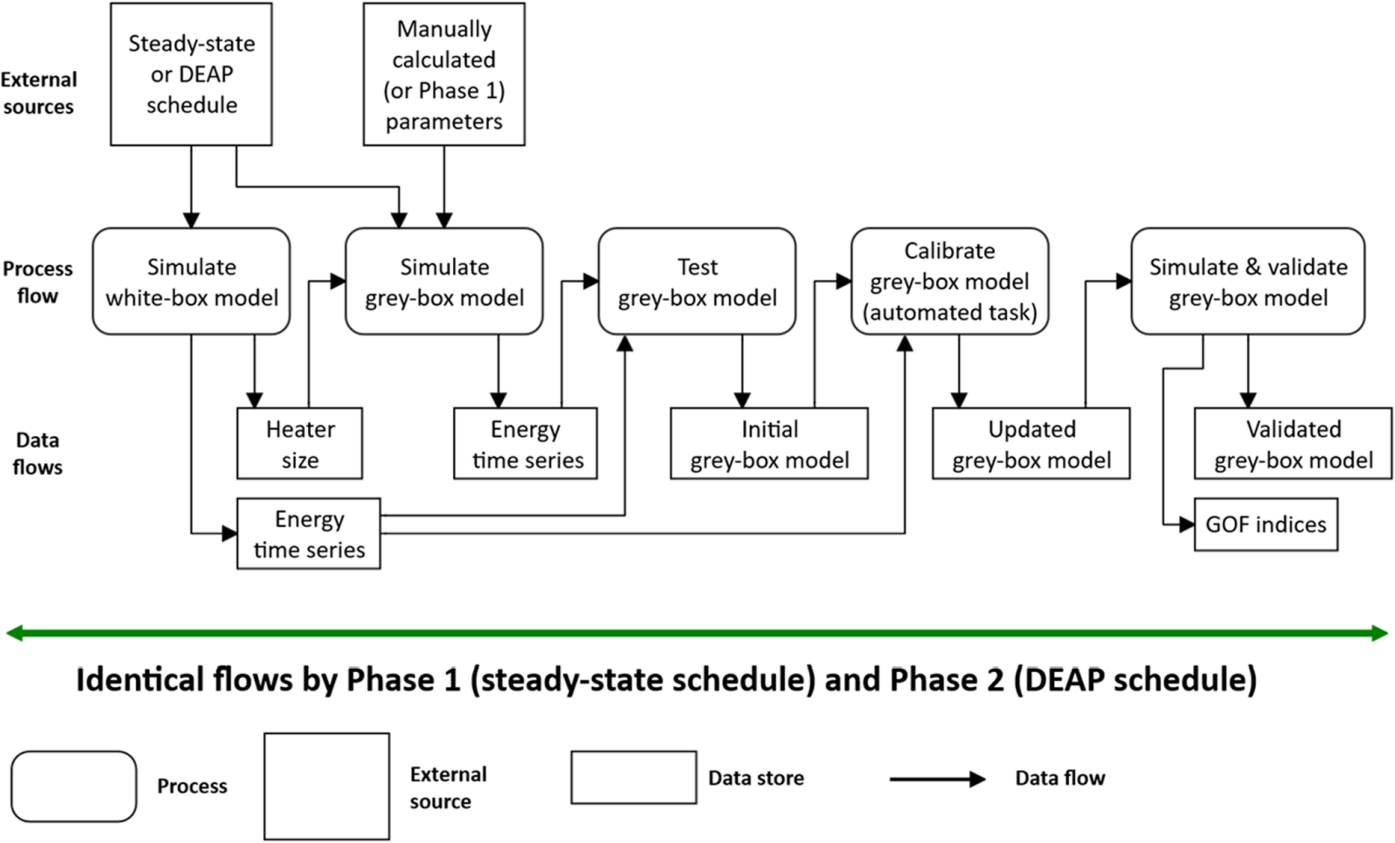
Calibration procedure and data flow. Identical flows used during both Phase 1 and Phase 2 but differentiated by energy-usage data from different heating schedules

To achieve both validations, the grey-box model calibration procedure comprised two phases. Both phases followed an *identical* data flow (Fig. 6), but each phase calibrated the model against a *different* heating schedule:

- Phase 1: heat-energy usage from simulation under steady-state schedule and
- Phase 2: heat-energy usage from simulation under DEAP schedule.

Home heater sizes in the grey-box models were estimated during previous simulations of the white-box models (Beagon et al., 2020). During calibration procedure, the simulations used the heaters to maintain a 20ºC setpoint adopted from TABULA (2013). Before the first simulation of Phase 1, the grey-box models were initialised with manually calculated values of tunable parameters. After the first simulation, the resulting monthly heat-energy usage was tested against that of a white-box model simulated under the same schedule. As expected, simulations with initial parameter values failed validation, and Phase 1 progressed to the automated task labelled “calibrate grey-box model” (Fig. 6).

After the automated calibrate task, the results were used to update the tunable parameters of the grey-box models. The updated models were subsequently re-simulated under both steady-state and DEAP schedules. Each simulation of an updated model calculated its monthly heat-energy usage per schedule. Those results were compared to the monthly results from simulating white-box models under identical schedules. The validation results of as-built and retrofit models appear in Table 4 and Table 5 respectively.

**Table 4:** Validation goodness-of-fit indices of calibrated as-built models using monthly heat-energy usage under two heating schedules: (1) steady-state and (2) DEAP

Label	Phase	Steady-state schedule		DEAP schedule	
		CV(RMSE) (%)	NMBE (%)	CV(RMSE) (%)	NMBE (%)
TH03	1	2.51	− 0.24	8.08	3.56
TH06	1	0.88	− 0.02	4.70	3.54
TH07	1	2.55	− 0.47	6.46	4.31

**Table 5:** Validation goodness-of-fit indices of calibrated retrofit models using monthly heat-energy usage under two heating schedules: (1) steady-state and (2) DEAP

Label	Phase	Steady-state schedule		DEAP schedule	
		CV(RMSE) (%)	NMBE (%)	CV(RMSE) (%)	NMBE (%)
TH03	1	3.31	− 0.07	9.47	8.25
TH06	1	1.48	0.24	22.10	15.11
TH07	1	5.19	4.01	19.04	13.31
TH03	2			3.75	− 2.70
TH06	2			7.06	1.76
TH07	2			6.50	0.85

As mentioned, the validation criteria were CV(RMSE) < 15%, and -5% < NMBE < 5%. Since Phase 1 validation failed in the case of retrofit models, Phase 2 became necessary. Phase 2 calibrated grey-box models, initialised with Phase 1 parameters values, to fit monthly heat-energy usage under the DEAP schedule.

Phase 1 calibration was sufficient for models of as-built archetypes (Table 4). In contrast, models of retrofit archetypes required Phase 2 calibration to achieve validation. The CV(RMSE) and NMBE validation indices improve significantly after Phase 2 as percentage values (Table 5). Note that retrofit archetypes must achieve smaller validation errors in terms of energy values, because retrofitted homes typically use less heat energy.

### Data sources for simulations

With the RC values of the building fabric subsystem now known, the modelling effort focused on the subsystem controlled by occupants: *the internal thermal zone*. Different occupant-operated setpoints and heating patterns result in different heat-energy usage calculated by simulations. Large variations in heat-energy usage are expressed as statistical distributions — a key result of this study.

Recent literature provided empirical sources to enable simulation of heating patterns and setpoints (Table 6). A review of gas or oil-fired central heated homes found thermostat settings from 13.1 to 27.3ºC — an “enormous” range (Shipworth et al., 2010). Although the mean and median temperatures were both 21ºC, the sample data was positively skewed with 40% at or above 22ºC.

**Table 6:** Empirical data sources of weather, home archetypes, household heating and occupancy. Descriptions of household data include sample size, location, and home type

Data type	Sample size (n)	Location	Home type	Source
IWEC2	Not applicable	Dublin Airport, Ireland	Not applicable	NREL, 2018
Home archetypes	Not applicable	Dublin, Ireland	Semi-detached and terraced	Badurek et al., 2014
Heating setpoints	49 retrofit, 62 as-built	Plymouth, England	Flats, semi-detached and terraced	Jones et al., 2016
Heating pattern types	Weekday: 43 retrofit, 75 as-built Weekend: 40 retrofit, 74 as-built	Plymouth, England	Flats, semi-detached and terraced	Jones et al., 2016
Heating times	275 (silent on retrofit state)	England nationally	Detached, flats, semi-detached and terraced	Huebner et al., 2015
Occupancy times	32 as-built	London, England	Flats	Zahiri & Elsharkawy, 2018

Social housing in England displayed large variations in heating setpoints, even among 62 as-built and 49 retrofit homes analysed (Jones et al., 2016). For instance, occupants in retrofit homes chose setpoints from a distribution of higher temperatures ($\overline{x }$ = 21.7ºC, std dev. = 3.2ºC, *n* = 49) than occupants of as-built homes ($\overline{x }$ = 20.2ºC, std dev. = 3.3ºC, *n* = 62). Notably, the same research referred to “thermal upgrade” of wall insulation as opposed to “retrofits”, indicating that primary heat generation (probably boilers) remained unchanged.

Considering the variation in occupant heating preferences, this study adopted setpoint temperatures, heating durations and schedules from available empirical sources, that themselves differ in sample size, home type and location (Table 6). In addition to setpoint distributions, three heating patterns emerged in social housing (Jones et al., 2016). “Double” (morning and evening) was the most common (45.9%), followed by “single” (24.3%) and “constant” 24 h (20.3%). Triple heating periods occurred in under 10% of weekdays and thus were neglected by this study.

Occupants also require ventilation to maintain good health. As explained in the companion paper (Beagon et al., 2020), the simulations maintained the minimum 0.4 ACH recommendation during occupancy periods (Ramos et al., 2015; Wargocki, 2018). Where lower levels of air permeability are achieved, Part L regulations require purpose provided ventilation (DECLG, 2011). Therefore, the simulations assumed that retrofit homes operated demand controlled ventilation (DCV) simultaneous with occupant-caused internal gains.

**Fig. 7:**
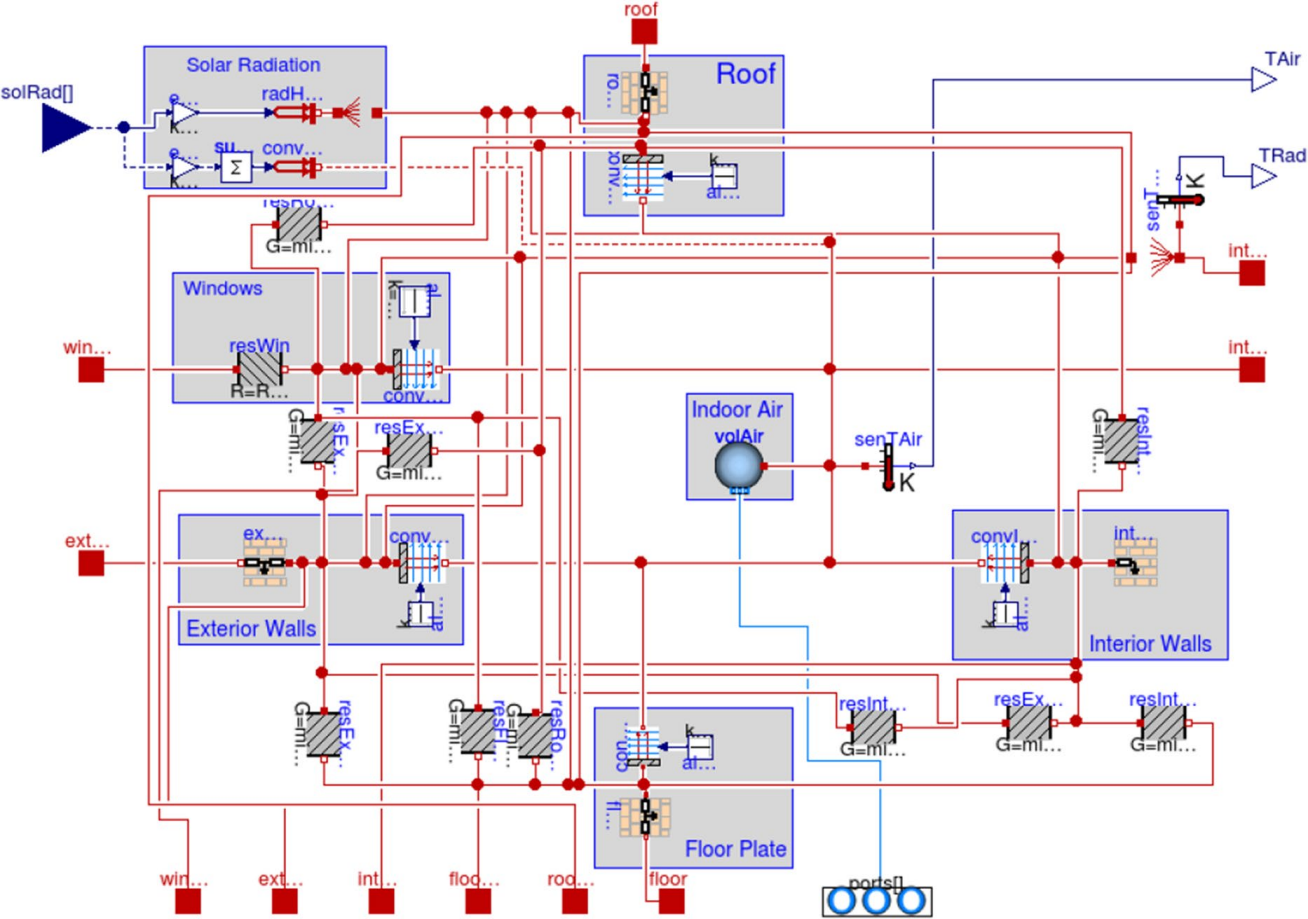
Modelica model of four building elements enclosing one indoor thermal zone labelled “Indoor Air volAir” (IEA Annex 60, 2017)

Finally, heating and ventilation operated upon a single internal zone within all models. This study’s AixLib model of a single zone building appears in (Fig. 7); it displays the four building elements of exterior walls, interior walls, floor plate and roof (IEA Annex 60, 2017). A proportional-integral-derivative (PID) controller, recommended in the AixLib library (RWTH EBC, 2020), tracked the temperature setpoint of the internal zone. Controller parameters enabled activation of heating when internal air temperature dropped 0.5ºC below setpoint, accompanied by limitation of short cycling by one-minute off-time. This controller was easily configured, closely tracked every setpoint, and limited short cycling (where a heat generator turns on and off too frequently).

### Heating and internal gains schedules

As listed in Table 6 data sources, research on 275 homes throughout England plotted four heating schedules as daily time-series: “Steady rise”, “Flat line”, “Two peak” and “Steep rise” (Huebner et al., 2015). Simulation heating times were extracted from the time-series plots by Huebner et al., (2015) and other research (Jones et al., 2016). Both sources lengthened weekend heating durations which this study also incorporated into simulation schedules (Table 7).

**Table 7:** Heating patterns schedules by time of day and hours (h) duration. Each pattern comprises four schedules, split by as-built and retrofit state, and weekday and weekend. Times adapted from Huebner et al., 2015; Jones et al., 2016

Pattern	As-built		Retrofit	
	Weekday	Weekend	Weekday	Weekend
Constant	00:00–24:00 (24 h)	00:00–24:00 (24 h)	00:00–24:00 (24 h)	00:00–24:00 (24 h)
Single	15:30–20:25 (4.9 h)	15:15–20:35 (5.4 h)	13:20–21:45 (8.4 h)	13:15–21:50 (8.6 h)
Double	06:40–08:10 & 16:40–20:35 (5.4 h)	07:00–09:00 & 16:30–20:35 (6.1 h)	06:40–08:10 & 16:20–20:50 (6.0 h)	07:00–09:00 & 16:20–20:30 (6.2 h)

The Steep rise time-series plot increased temperature from the morning throughout the day, peaking “before 21:00”, specifically 20:30 in its diurnal plot (Huebner et al., 2015). Since Steep rise descended to the lowest temperature of 16ºC among all four time-series plots, it resembled the single heating pattern identified by Jones et al. (2016). Occupants significantly increased the duration of the single heating pattern after retrofit, compared to the double pattern. Durations of the *single* heating pattern were lengthened: weekdays 4.9 h to 8.4 h, and weekends 5.4 h to 8.6 h (Table 7). Weekday and weekend durations of the *double* pattern lengthened marginally after retrofit: weekday 5.4 h to 6.0 h, and weekend 6.1 h to 6.2 h.

Longer heating durations after retrofit indicated that the occupants had been rationing their home heating. While occupants ought to be comfortable in their homes, the lengthening of heating durations and other “comfort taking” may have reduced or negated any savings in heat energy. Occupant “comfort taking” and the resulting rebound effect and potential “backfire” have been outlined in the Introduction.

Finally, internal gains caused by occupants were already calculated (Beagon et al., 2020), and their schedules lengthened at weekends for the single and double heating patterns in Table 8. Schedules of internal gains under the constant and single heating patterns were derived from the dominant occupancy patterns defined for a London tower block by Zahiri and Elsharkawy (2018). The dominant occupancy patterns emerged from 32 responses to the 108 questionnaires distributed to every tower block flat. This sample size of 32 exceeded the 25 necessary for valid correlation tests according to Oppenheim (1992), whom Zahiri & Elsharkawy cited as justification.

**Table 8:** Simulated internal gains schedules by heating pattern. Schedules apply to both as-built and retrofit home archetypes

Pattern	Weekday	Weekend
Constant	08:00–22:00	08:00–22:00
Single	06:30–22:00	08:30–22:30
Double	06:40–08:30 & 16:20–22:15	07:00–10:30 & 15:30–22:30

Regarding the double heating pattern, Marshall et al. (2016) provided detailed times of internal gains by an applicable “Working Family”. This presented study used a double heating pattern where the morning heating coincided with internal gains (Table 7, Table 8). At the weekend, the double heating pattern and associated internal gains did not start until 07:00, as extracted from time use surveys (Buttitta et al., 2017).

### Characterising heat-energy distributions

With the heating patterns and internal gains defined (Table 7 and Table 8), simulations then calculated heat-energy usage across two ranges of heating setpoints. As described in section Data sources for simulations, setpoint distribution parameters differed between as-built ($\overline{x }$ = 20.2ºC, std dev. = 3.3ºC) and retrofit homes ($\overline{x }$ = 21.7ºC, std dev. = 3.2ºC). An R function used the parameters of the two setpoint distributions to randomly generate separate sets of as-built or retrofit setpoints. Normal distributions were assumed. Each of the two sets contained 300 sample setpoints (*n* = 300), to enable distribution fitting (Keller, 2011). Note that *n* = 300 did *not* imply that each neighbourhood comprises over 300 similar homes.

The 300 as-built setpoints were applied to all as-built models, then simulated under the as-built schedules of each heating pattern. Similarly, the 300 retrofit setpoints were applied to all retrofit models, then simulated under the retrofit schedules of each heating pattern. A total of 18 models represented every combination of three archetypes, three heating patterns, and two states of as-built and retrofit. Thus, the 18 models were each simulated under 300 heating setpoints to produce 5400 values of yearly heat-energy usage.

All 300 simulations of an archetype and heating pattern combination, modelled in either as-built or retrofit state, calculated one value of heat-energy usage. After the simulations, the 300 usage values formed a dataset representing that archetype, heating pattern and state. Each energy-usage dataset was itself a distribution, characterised by sample statistics of mean ($\overline{x }$) standard deviation (s) and skewness ($\gamma$). Skewness, a dimensionless statistic, indicates a distribution’s shape and symmetry. A perfectly symmetrical distribution was measured at zero skewness. Positive or negative skewness values indicated a longer right or left tail respectively. Multiple skewness formulas existed in literature (Tabor, 2010; Versluis & Straetmans, 2015); the former publication defined 11 skewness formulas and the latter 14. This study adopted the “formal” or “textbook” formula; the Fisher-Pearson third moment coefficient of skewness (3), unadjusted due to the large sample *n* = 300 (Joanes & Gill, 1998; NIST, 2013).

3 $$\mathrm{Skewness}:\gamma=\frac{m_3}{\sqrt{m_2^3}}\;\left[-\right]\;\mathrm{where}\;m_r=\frac1n\sum(x_i-\overline x)^r$$

A highly skewed distribution requires $\gamma$ > 1, a moderately skewed distribution 0.5 $\le$ $\left|\gamma \right| \le$ 1, and an approximately or “fairly” symmetrical distribution $\left|\gamma \right|$ < 0.5 (Brown, 2016; Bulmer, 1979). All calculations and plots were executed in R statistical language. Function skewness(*x,* type = 1) calculated the textbook skewness (Meyer, 2020), and ggplot2 functions plotted the graphs (Beagon, 2020; Wickham, 2016).

As introduced in section Statistical distributions of home energy usage, the Anderson–Darling (AD) technique was selected to test fit of the heat-energy datasets to statistical distributions. The AD test adopted a null hypothesis that sample values arose from a common statistical distribution, specified by name or as another dataset. Smaller AD statistics and larger *p*-values indicated no evidence of deviation from the hypothesised common distribution. Thus, AD test statistics *reduced* with increasing goodness-of-fit (GOF) with the hypothesised common distribution. This study uses the AD test function within the R kSamples package (Scholz & Zhu, 2019). As a kSamples function, it tested the hypothesis that k number of independent samples arose from a common continuous distribution.

## Results

### Statistics of heat-energy usage

The heat-energy usage statistics of all nine combinations of heating pattern and home archetype appear in Table 9. Only five of the combinations, however, show decreased mean heat-energy usage after retrofit. Four combinations increased mean heat-energy usage, including all three TH07 heating patterns. Among the *shallow* TH07 retrofits, heat-energy savings by building fabric upgrades were counteracted by higher occupant heating demands.

**Table 9:** Heat-energy usage by combination of archetype and heating pattern, in as-built and retrofit states. Sample statistics comprise the mean ($\overline{x }$), standard deviation (s) and skewness ($\gamma$)

Pattern	Label	As-built			Retrofit		
		$\overline{x }$ (kWh)	s (kWh)	$\gamma$	$\overline{x }$ (kWh)	s (kWh)	$\gamma$
Constant	TH03	9654	4236	0.462	4437	2151	0.507
Constant	TH06	11,553	4653	0.415	6201	2637	0.423
Constant	TH07	6215	2913	0.502	6992	2686	0.363
Single	TH03	5079	2371	0.510	3155	1667	0.601
Single	TH06	4184	1878	0.697	4357	1798	0.411
Single	TH07	3035	1656	0.696	4050	1789	0.490
Double	TH03	7016	2804	0.345	3903	1694	0.382
Double	TH06	7275	2656	0.382	4805	1848	0.355
Double	TH07	4893	2110	0.426	5328	1881	0.303

Table 10 displays the decreases and increases in mean heat-energy usage as positive and negative savings respectively. Paired *t*-tests were performed on each combination’s heat-energy usage between their as-built and retrofit states. The null hypothesis of no difference between as-built and retrofit states was rejected with a *p*-value below 0.001 for all combinations of archetype and heating pattern.

**Table 10:** Savings in mean heat-energy usage by combination of archetype and heating pattern. The mean value is accompanied by its 95% confidence interval (CI)

Heating pattern	Archetype label	Mean heat-energy saving (kWh)	CI of mean heat-energy saving (kWh)
Constant	TH03	5217	4981, 5454
Constant	TH06	5352	5213, 5581
Constant	TH07	− 777	− 805, − 747
Single	TH03	1924	1843, 2004
Single	TH06	− 173	− 192, − 153
Single	TH07	− 1014	− 1034, − 996
Double	TH03	3113	2987, 3239
Double	TH06	2470	2397, 2562
Double	TH07	− 436	− 463, − 408

Based on the mean values in Table 10, the single heating pattern fails to save heat energy in archetype TH06, and the aforementioned TH07. An explanation is that occupants extend the weekday duration of the single heating pattern by three and half hours after retrofit (section Heating and internal gains schedules). The short duration of the single pattern, however, means that it minimises the heat-energy usage by *all* archetypes in both as-built and retrofit states.

### Exploring distributions of heat-energy usage

Having calculated the statistics from each dataset of heat-energy usage, the datasets were then analysed as probability density distributions. As mentioned, each dataset of 300 energy-usage values represents one combination of archetype and heating pattern in as-built or retrofit state. These distributions were explored using quantile–quantile plots, goodness-of-fit (GOF) evaluation and hypothesis testing.

### Quantile–quantile plots

A quantile–quantile (QQ) plot is an initial exploration of a dataset’s fit to normality. All QQ plots appear in this paper’s Supplementary Material. The plots display a dataset of heat-energy usage, alongside the theoretical normal distribution parameterised from energy-usage dataset. Every plot’s horizontal axis displays Z-scores representing the distance between an energy-usage value and the dataset mean. Since the distance is expressed in standard deviations, the range from − 3 to + 3 captures 99.7% of the theoretical normal distribution. All QQ plots approximate to normality at central values but diverge at their tails. The largest proportional divergence at the tails manifests under the single heating pattern.

### Goodness-of-fit to normal distributions

The Anderson Darling (AD) technique, described in section Characterising heat-energy distributions, was used to test the fit between two 300-sample datasets. The first dataset contained the simulation heat-energy usage and the second dataset contained 300 quantiles of the theoretical normal distribution. The mean and variance parameters of that theoretical normal distribution were calculated from the simulation dataset. Results of the AD tests appear in Table 11, comprising the test statistics and the *p*-values. Despite deviations from normality seen in the QQ plots, there is insufficient evidence to reject the null hypothesis of normality at a standard *p*-value of 0.05.

**Table 11:** Anderson–Darling tests of fit between heat-energy usage datasets and normal distribution datasets. Results comprise test statistics and asymptotic *p*-values. All sample sizes were *n* = 300

Pattern	Label	As-built		Retrofit	
		AD statistic	*p*-value	AD statistic	*p*-value
Constant	TH03	0.671	0.585	0.807	0.476
Constant	TH06	0.515	0.732	0.538	0.709
Constant	TH07	0.769	0.504	0.413	0.836
Single	TH03	0.914	0.405	1.180	0.273
Single	TH06	1.500	0.175	0.509	0.737
Single	TH07	1.590	0.157	0.759	0.512
Double	TH03	0.414	0.835	0.534	0.712
Double	TH06	0.494	0.753	0.401	0.848
Double	TH07	0.605	0.644	0.334	0.911

### Goodness-of-fit to Weibull distributions

Weibull distributions, introduced in section Statistical distributions of home energy usage, have been used to represent home energy use at different spatial scales (Irwin et al., 1986; Munkhammar et al., 2014). Furthermore, the Weibull distribution is the most popular model of wind energy evaluation (Wais, 2017). Wind energy analysts often estimate Weibull parameter by the “graphical method”, that is mathematically similar to a linear regression technique.

Applying the graphical method to the energy-usage datasets enabled estimation of the Weibull parameters of shape and scale. Shape and scale parameters were estimated for each combination of archetype and heating pattern, in both as-built and retrofit states (Table 13). The larger AD *p*-values in both as-built and retrofit states (Table 13) indicate that heat-energy distributions fit closer to Weibull distributions compared to normality (Table 11).

### Hypothesis test that mean heat-energy usage equals an official estimate

The hypothesis test was a one sample *t*-test against a population average (μ). It tested the null hypothesis that the difference between each distribution of heat-energy usage and μ = 4600 kWh was zero. The 4600-kWh value reflects the SEAI estimate that a terraced, “efficient” home demands 4.6 MWh/year heat energy (SEAI, 2015). Confidence intervals (CI) were calculated at 95% for all combinations of archetypes and heating patterns, in as-built and retrofit state (Table 12).

**Table 12:** Mean ($\overline{x }$) and 95% confidence interval (CI) of heat-energy usage, by combination of archetype and heating pattern in as-built and retrofit states. The *p*-value results from the hypothesis that mean heat-energy use equals 4600 kWh

Archetype label	Heating pattern	As-built		Retrofit	
		$\overline{x }$ and CI (kWh)	*p*-value	$\overline{x }$ and CI (kWh)	*p*-value
TH03	Constant	9654 (9173, 10 136)	< 0.001	4437 (4192, 4681)	0.190
TH03	Single	5079 (4810, 5348)	< 0.001	3155 (2966, 3345)	< 0.001
TH03	Double	7016 (6697, 7334)	< 0.001	3903 (3710, 4095)	< 0.001
TH06	Constant	11 553 (11 025, 12 082)	< 0.001	6201 (5901, 6500)	< 0.001
TH06	Single	4184 (3971, 4397)	< 0.001	4357 (4152, 4561)	0.020
TH06	Double	7275 (6973, 7577)	< 0.001	4804 (4594, 5015)	0.056
TH07	Constant	6215 (5884, 6546)	< 0.001	6992 (6686, 7297)	< 0.001
TH07	Single	3035 (2847, 3224)	< 0.001	4050 (3847, 4254)	< 0.001
TH07	Double	4893 (4653, 5133)	0.017	5328 (5115, 5542)	< 0.001

In all but two combinations of retrofit archetype and heating pattern, *p*-values show sufficient evidence to reject the null hypothesis at the 5% significance level. In conclusion, the null hypothesis that mean heat-energy usage equals 4600 kWh is rejected by all archetypes in as-built state, and seven of the nine archetypes in retrofit state, at the 0.05 significance level.

## Discussion

### Discussion of heat-energy statistics

The statistics of the heat-energy usage are now discussed in the sequence of constant, single and double heating patterns.

### Constant heating pattern

As expected, the largest savings in heat-energy usage attributable to retrofit were achieved under the constant heating pattern. Within the constant pattern, the mean values of heat-energy usage decreased under deeper retrofits of archetypes TH03 and TH06 but increased after the shallow retrofit of TH07, causing positive and negative energy savings respectively (Table 10). Thus, the retrofit of TH07 actually “backfired” by actually increasing the archetype’s mean heat-energy usage. As described in the Introduction, cases where occupant “comfort taking” exceeded savings in energy usage were termed “backfire” by (Druckman et al., 2011). Specific to the constant pattern, the TH07 backfire entailed a 10% increase in mean (95% CI) heat-energy usage: from 6215 (5884, 6546) kWh to 6992 (6686, 7297) kWh (Table 12).

Under the constant heating pattern, variations of all archetypes’ heat-energy usage reduced from as-built to retrofit state. The variations, expressed as ranges of standard deviation (s), reduced from asbuilt state 2913–4653 kWh to retrofit state 2151–2686 kWh (Table 9). As expected, the largest reductions in standard deviation were achieved by deeper retrofits of older archetypes: TH03 − 2085 kWh and TH06 − 2016 kWh, whereas the shallow retrofit of the youngest archetype TH07 reduced standard deviation by merely − 227 kWh.

Despite their reductions in variation after retrofit, the heat-energy distributions of TH03 and TH06 slightly increased positive skew. Retrofit TH03 entered the moderate skewness range 0.5 $\le$ $\left|\gamma \right| \le$ 1. The as-built and retrofit skewness ranges were 0.415–0.502 and 0.363–0.507 respectively (Table 9). In Appendix Fig. 9, heat-energy distributions under the constant pattern displayed the manifestations of positive skew — extending right-hand tails over high values and diverging from the symmetry that characterises a normal distribution.

### Single heating pattern

The smallest heat-energy savings were achieved under the single heating pattern, because of the pattern’s longer heating durations after retrofit (section Heating and internal gains schedules). Under the single pattern, only the deepest retrofit of archetype TH03 delivered savings in mean heat-energy usage. Archetypes TH06 and TH07 actually increased heat-energy usage, shown as negative savings (Table 10). A shallow retrofit of TH07, compounded by longer and warmer heating durations after retrofit, resulted in the study’s largest retrofit backfire. Specific to the single pattern, the TH07 backfire entailed a one-third increase in mean (95% CI) heat energy: from 3035 (2847, 3224) kWh to 4050 (3847, 4254) kWh (Table 12).

Under the single heating pattern, the smallest reductions in variation from as-built to retrofit state occurred. Variations, expressed as ranges of standard deviation, reduced from as-built state 1656–2371 kWh to retrofit state 1667–1798 kWh (Table 9). Within the pattern, retrofit of the oldest archetype TH03 again achieved the largest reduction in standard deviation, from 2371 to 1667 kWh.

In Appendix Fig. 10, heat-energy distributions under the single pattern displayed the variation and skewness across archetypes. Overall, the distributions’ skewness ranges in the as-built state (0.510–0.696) or retrofit state (0.411–0.601) were moderately skewed or mainly “fairly” symmetrical respectively (Table 9). However, the skewness of the TH03 distribution increased from 0.510 to 0.601, although remained within the moderate range 0.5 $\le$ $\left|\gamma \right| \le$ 1, while the younger TH06 and TH07 archetypes reduced their distribution skewness. The post-retrofit increase in TH03 skewness reflected the non-linear increases in heat-energy usage to maintain the highest heating setpoints.

### Double heating pattern

Energy savings achieved under the double heating pattern exceeded those under the single pattern, but undershot those under the constant pattern (Table 10). Like the constant heating pattern, savings in mean heat-energy usage are achieved by deeper retrofits of older archetypes TH03 and TH06. The shallow retrofit of TH07 consistently failed to save heat energy under all heating patterns. Specific to the double pattern, the TH07 backfire entailed a 9% increase in mean (95% CI) heat energy: from 4893 (4653, 5133) kWh to 5328 (5115, 5542) kWh (Table 12).

The variations in heat-energy distributions, expressed as ranges of standard as-built deviation, reduced from as-built state 2110–2804 kWh to retrofit state 1694–1881 kWh (Table 9). As expected, retrofit of the oldest archetype TH03 achieved the largest reduction in standard deviation from 2804 to 1694 kWh.

In Appendix Fig. 11, heat-energy distributions under the double pattern displayed the variation and skewness across archetypes. All archetype distributions met the “fairly” symmetrical definition where 0.0 $\le$ $\left|\gamma \right| \le$ 0.5. The as-built and retrofit skewness ranges were 0.345–0.426 and 0.303–0.382 respectively (Table 9). These variation and skewness statistics manifested in heat-energy distributions that resemble normal distributions and peaked over mean values, especially after retrofit (Fig. 11).

### Discussion of heat-energy distribution fitting

The fit of the heat-energy distributions with normal and Weibull distributions are now discussed.

### Quantile–quantile plots

It is important to note that all heat-energy usage results are, by their nature, positive. Therefore, positive values of heat-energy usage must diverge from any negative values extrapolated by the *lowest* Z-scores of a QQ plot. The QQ plots assumed a *theoretical* normal distribution. For instance, as-built QQ plots under the constant pattern extended into negative values because of large standard deviations with the energy-usage datasets. Note that standard deviation is both a measure of dispersion, and a parameter of the theoretical normal distribution. To summarise, as-built heat-energy usage appeared to diverge further from normality, because of their larger standard deviations compared to retrofit heat-energy usage.

### Exploring the goodness-of-fit to normal distributions

The QQ plots displayed evidence of divergence from normality at the distribution tails. While it remained relatively small, the divergence was also reflected in distribution statistics. For instance, as-built archetypes heated under the single pattern resulted in the largest AD statistic (Table 11), and the most skewed plots (Appendix Fig. 9).

All AD test *p*-values were too large to reject the null hypothesis of a common normal distribution at the significance value of 0.05. However, as seen in the QQ plots, there were deviations from normality and the *p*-values were inconsistent, ranging from 0.157 to 0.911. Therefore, AD tests were repeated in case each dataset fitted better with its theoretical Weibull distribution.

### Exploring the goodness-of-fit to Weibull distributions

Weibull shape parameters range from 1.94 to 3.22 (Table 13), and a majority of 13 achieved the log-normal distribution criteria of shape value $\ge$ 2.5 (NCSS LLC, 2019). The double heating pattern was the only pattern that was consistently expressed as a log-normal distribution of heat-energy usage. Nonetheless, the pattern’s highest shape value of 3.22 revealed a significant shortfall below the 3.6 value of a normal distribution.

**Table 13:** Heat-energy usage expressed as Weibull parameters accompanied by the goodness-of-fit *p*-value to the Weibull distribution. The Anderson–Darling (AD) test quantifies the goodness-of-fit *p*-value, while CV(RMSE) and NMBE indices quantify the goodness-of-fit error

Archetype label	Heating pattern	As-built					Retrofit				
		$\mathrm{Shape}$	Scale (kWh)	AD *p*-value	CV(RMSE) (%)	NMBE (%)	$\mathrm{Shape}$	Scale (kWh)	AD *p*-value	CV(RMSE) (%)	NMBE (%)
TH03	Constant	2.51	10 817	0.998	2.85	0.232	2.20	4985	0.999	2.49	0.144
TH03	Single	2.35	5694	0.997	3.05	0.305	1.96	3550	0.999	3.00	− 0.104
TH03	Double	2.77	7844	0.999	2.34	0.160	2.50	4378	0.999	2.29	0.153
TH06	Constant	2.77	12 910	0.993	2.88	0.195	2.58	6947	0.999	2.54	0.170
TH06	Single	2.62	4666	0.542	6.29	0.535	2.67	4878	0.996	2.75	0.141
TH06	Double	3.14	8088	0.908	3.37	0.206	2.89	5364	0.993	2.66	0.137
TH07	Constant	2.30	6977	0.999	2.61	0.194	2.92	7800	0.993	2.69	0.168
TH07	Single	1.94	3402	0.999	3.55	0.233	2.51	4535	0.991	3.16	0.293
TH07	Double	2.54	5483	0.999	2.65	0.192	3.22	5921	0.972	2.72	0.148

While four of the largest shape parameters manifest under the double heating pattern, a well-defined association between shape parameters and heating patterns fails to appear. Retrofitting the archetypes does not consistently reduce, increase or transform the shape parameters of heat-energy distributions. As expected, Weibull scale parameters reduce after retrofit, due to the savings in heat-energy usage.

The goodness-of-fit achieved by each Weibull distribution is measured by model calibration indices: CV(RMSE) and NMBE. Almost universally, these indices improve slightly after retrofit. All heating combinations, as-built and retrofit, achieve both model calibration standards: CV(RMSE) $\le 5\%$ and $-5\mathrm{\%}\le$ NMBE $\le 5\%$. Weibull distributions can be seen to maximise these fitting errors at their plot peaks (Appendix Figs. 9, 10 and 11).

### Hypothesis that mean heat-energy usage equals an official estimate

**Fig. 8:**
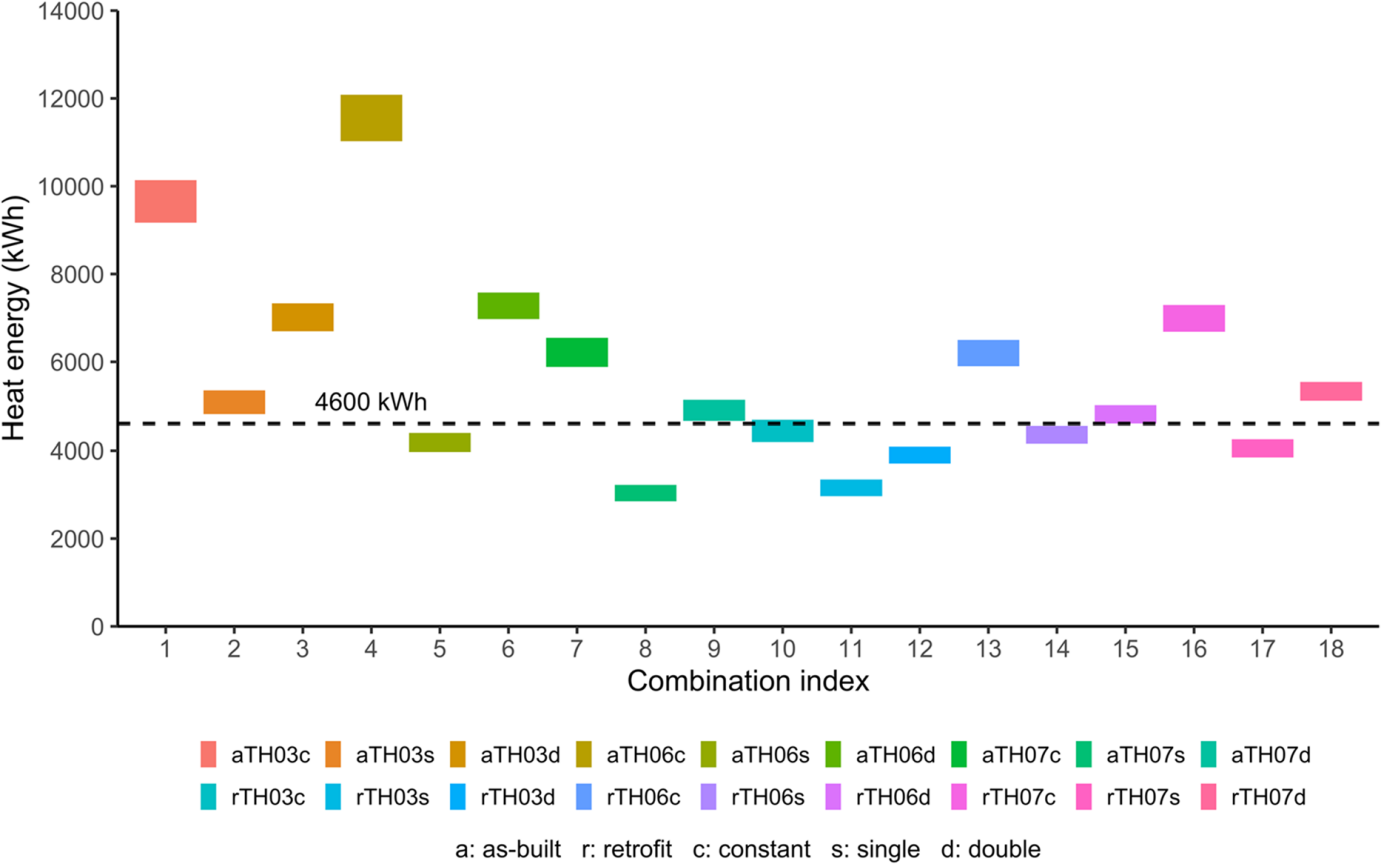
Mean (95% CI) of heat-energy usage by combinations of archetype and heating pattern in as-built and retrofit states. Across the three heating patterns, retrofit TH06 approaches the 4600-kWh official estimate (dashed line)

**Fig. 11:**
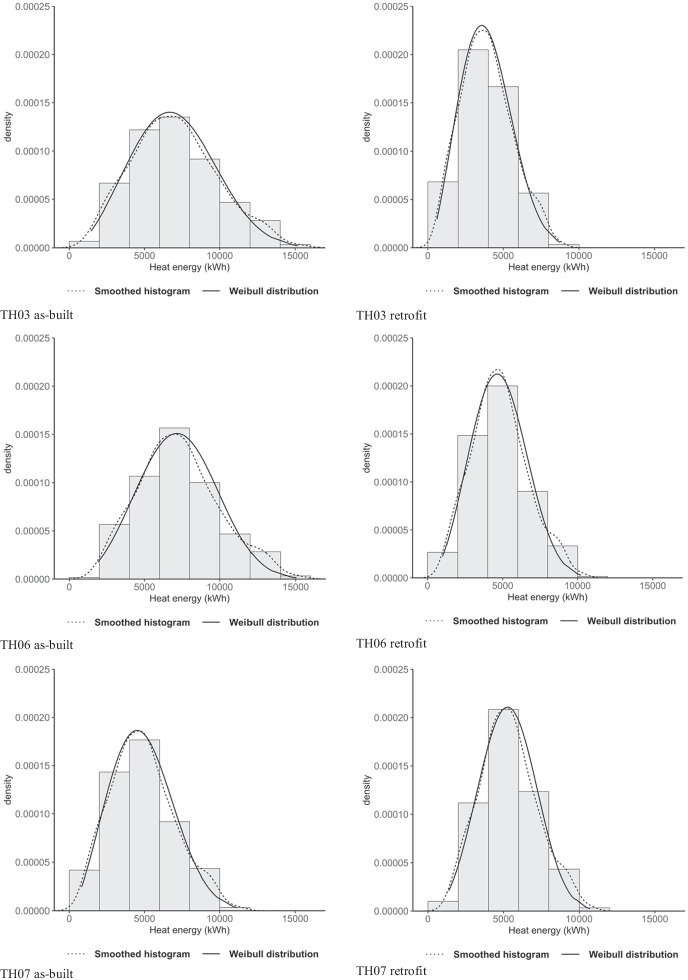
Distributions of yearly heat-energy usage by as-built and retrofit archetypes under *double* heating pattern. The three distributions are histogram bars, smoothed histogram density and Weibull density

Finally, the discussion reviews which one of the mean values of heat-energy usage approaches the official estimate. All the confidence intervals (CI) are *disjointed*, highlighting how occupants cause *different* mean values of heat-energy usage in the *same* home archetype. Most of the CIs exclude the official 4600-kWh estimate of mean heat-energy usage (Fig. 8).

Two combinations in *retrofit* state contain 4600 kWh within their mean 95% CIs: TH03 constant heating pattern 4192–4681 kWh, and TH06 double heating pattern 4594–5015 kWh (Table 12). After retrofit, the semi-detached TH06 reduces its heat energy to become the closest archetype to the 4600 kWh value. Under the constant, single and double patterns, retrofitted TH06 used three different means of heat energy: 6201 (5901, 6500) kWh, 4357 (4152, 4561) kWh and 4804 (4594, 5015) kWh.

Overall, archetypes in the retrofit state diverge less from the official 4600 kWh value, than those in the as-built state (Fig. 8). Considering all heating patterns, the retrofit TH06 archetype of a semi-detached house comes closest to the official estimate. Therefore, there is evidence that the official estimate is representative of “efficient” medium homes — especially retrofit semi-detached houses, as opposed to low-performing homes.

### Discussion of study limitations

Certain limitations existed in the models and in the predictive application of their results. Starting with the models themselves, the heating controller assumed heating setpoints were maintained throughout the year, even after the heating season. Occupants may have behaved otherwise, and completely deactivated space heating during the summer months.

While the Modelica models did incorporate increased air infiltration due to temperature differentials, they lacked a realistic response to wind speeds. In general, the models also assumed the correct installation and operation of demand controlled ventilation.

This study ignored uncommon occupant heating patterns, for example the triple heating pattern. A mass transition to working from home is covered by the constant heating pattern. Any evaluation of large-scale home energy retrofit must re-assess the popularity of heating patterns, especially for working families.

The weather file used by all simulations comprised selected days from previous years. Therefore, simulations exclude extreme weather conditions that are more likely in the future. Conventionally, homes located in cool temperate climates, such as Ireland’s, lacked built-in air conditioning. Certain climate change outcomes would make air-conditioning necessary for human comfort, necessitating a holistic reassessment of home energy usage.

## Conclusions

This paper defined a methodology to calibrate and realistically simulate grey-box models representing home heating. By incorporating occupant variables of heating setpoints and heating patterns, the methodology provides a distribution of heat-energy usage instead of a single value. Three case studies, of home archetypes in a cool temperate climate, demonstrated the results of the methodology.

Regarding the results of the methodology, all datasets of home heat-energy usage formed distributions that were positively skewed instead of normality. Positively skewed distributions cluster most values around their lower left tail, while the distribution’s right tail extends longer — pulled rightward by a few high values.

After retrofit however, most distributions were classified as fairly symmetric, having a skewness value below 0.5, with a minority remaining moderately skewed, having a skewness value between 0.5 and 1.0. There is no evidence of deviations from the Weibull distributions parameterised from the heat-energy dataset. However there exists, some evidence of deviation from log-normal distributions and evidence to reject fitting to normal distributions. Other remarks on the heat-energy usage distributions include:

- Heat-energy usage distributions are *different* between heating patterns of the *same* home archetype, both as-built and retrofit.
- Only deepest retrofit, implemented on the oldest archetype TH03, appears to consistently reduce the mean average of heat energy use.
- Retrofit of a higher performing home tends to increase the distribution symmetry but significant variance persists.
- These large variances in home heat-energy values require Weibull distributions that cannot extend into negative values.
- Positive skewness, albeit reduced, persists in the heat-energy usage of retrofitted homes.

Turning to the hypothesis that average heat-energy usage by an “efficient medium” home in Ireland equals a single value of 4600 kWh/year: this study finds evidence contrary to this hypothesis. Of the 18 heat-energy datasets, only two produced a mean value confidence interval that overlapped with the hypothesised average value. Two retrofit archetypes TH03 and TH06, each under a different heating pattern, produce a mean 95% confidence interval containing 4600 kWh/year. Since “double” is the most popular heating pattern, retrofit TH06 double heating pattern is the more likely source of a 4600 kWh/year average than retrofit TH03 under a constant heating pattern. TH06 is a semi-detached home constructed during 1978–1982, while TH03 is a terraced home constructed during 1930–1949.

### Implications and future work

The hypothesis results imply that an official estimate of heat demand by a medium-sized, efficient home archetype (SEAI, 2015), does *not* equal the mean heat-energy usage by most combinations of the studied home archetypes and heating patterns (Fig. 8). The reason is that heat-energy usage by each combination is itself a distribution, characterised by its own distinct average.

Evidence does exist that the estimated 4600 kWh of heat demand by efficient “medium” homes, defined by SEAI as terraced homes, is relevant. Heat-energy usage by retrofitted (and arguably efficient) archetype TH06 approaches the official estimate. However, TH06 represents a semi-detached home a home type that fits SEAI’s third, and final, category labelled “large” (SEAI, 2015).

In conclusion, evidence exists that the range of nine archetypes used by SEAI need expansion in order to accurately represent the Irish housing stock (SEAI, 2015). Higher resolution in home archetypes is needed, especially among the terraced and semi-detached homes that are popular in urban areas. Given their similar floorspace but different envelope, terraced and semi-detached homes warrant their own archetypes. Such archetypes should be further subdivided by as-built and retrofit state, to assist homeowners in selecting the optimum combination of fabric retrofit and heat pump. Elements of this proposed work will form part of SEAI’s National Heat Study: a “comprehensive assessment of the potential for efficient heating and cooling in Ireland” (SEAI, 2021).

Looking forward, work could extend from home archetype’s usage of heat energy to secondary energy. Research questions could be (1) what are the secondary-energy savings attributable to boiler upgrades or replacement by heat pumps? and (2) what is the energy usage across a neighbourhood of home archetypes before and after retrofit? Any new neighbourhood analysis would require new proportions of heating patterns, given the pandemic-induced shift to home working. Realistic distributions of secondary and primary energy usage — and savings attributable to *large-scale* home energy retrofit — could then be calculated.

### Electronic supplementary material

Below is the link to the electronic supplementary material.

Supplementary file1 (DOCX 2.41 MB)

## Figures and Tables

**Fig. 9:**
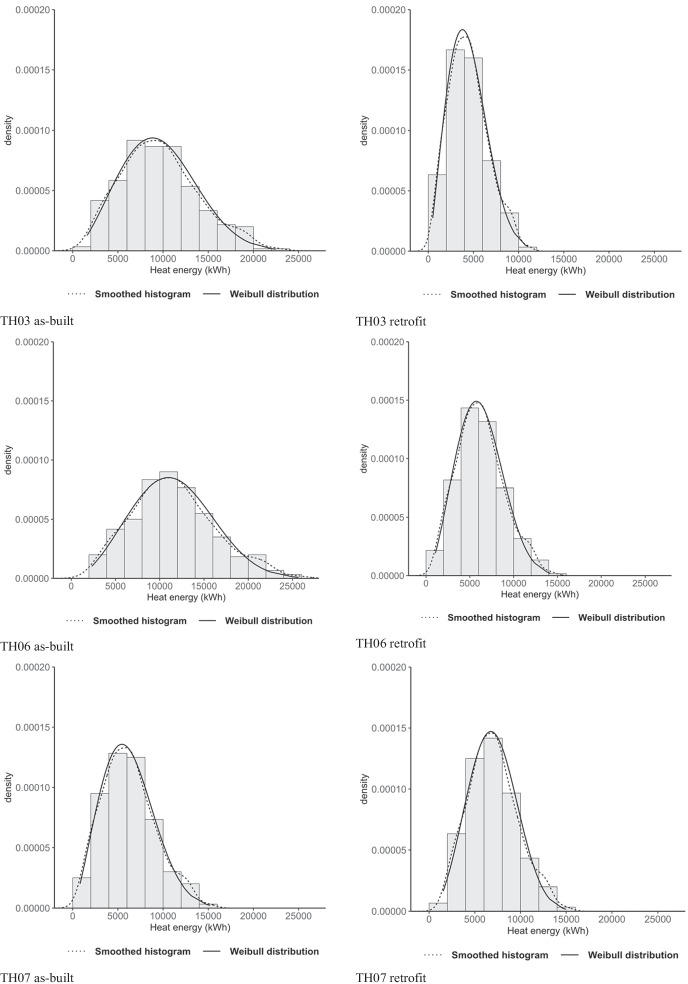
Distributions of yearly heat-energy usage by as-built and retrofit archetypes under *constant* heating pattern. The three distributions are histogram bars, smoothed histogram density and Weibull density

**Fig. 10:**
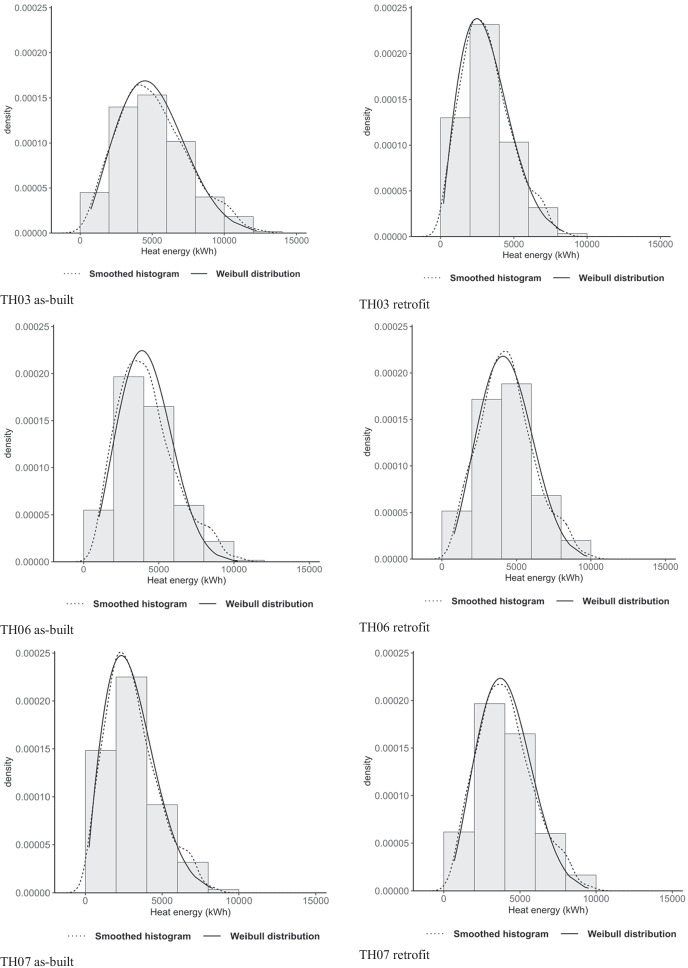
Distributions of yearly heat-energy usage by as-built and retrofit archetypes under *single* heating pattern. The three distributions are histogram bars, smoothed histogram density and Weibull density

## Data Availability

The datasets generated owned by Paul Beagon are available for publication if Journal wishes.

## Appendix. Distributions of heat-energy usage

The following distributions plots display how probability density distributions of heat-energy usage by home archetypes fit to Weibull distributions. Separate plots are presented for archetypes in both as-built and retrofit states. Heat energy is the energy type that warms homes’ internal air to setpoint temperatures at scheduled times. Occupants vary heating setpoints and schedules, thus varying heat-energy usage across the same archetype. Savings in heat energy are attributable to the fabric-only retrofits analysed in this study.

## Footnotes

1 American Society of Heating, Refrigeration and Air-Conditioning Engineers.
