# Supplementary material for: A grey-box modelling methodology to express home heat-energy usage as statistical distributions — case studies in urban Ireland
Source: Energy Effic. 2022 May 31;15(5):31. doi: 10.1007/s12053-022-10038-9 (PMC9156508; doi:10.1007/s12053-022-10038-9)
Supplement: Supplementary file 1 — Supplementary file1 (DOCX 2.41 MB) [file 12053_2022_10038_MOESM1_ESM.docx]

# Supplementary Material:

# Title: “A grey-box modelling methodology to express home heat-energy usage as statistical distributions — case studies in urban Ireland”

## Quantile-quantile plots of heat-energy datasets

Quantile-quantile (QQ) plots explore the fit to normality by the datasets of heat-energy usage. The heat-energy datasets contain the results produced by yearly simulation of home archetype models under different heating patterns. Heat energy is the energy type that warms a home’s internal air to maintain a setpoint temperature. Any savings measured by heat-energy usage, and attributable to home energy retrofit, were achieved by building fabric measures. (Energy savings achieved by improvements to a home’s heat generator are measured in secondary energy).

All QQ plots compare probability densities. Each plot displays a heat-energy dataset called “results”, alongside the theoretical normal distribution — a distribution parameterised using the “results” dataset. Every plot’s horizontal axis displays Z-scores representing the distance between a heat-energy result and its dataset mean. Since the Z-score distance is expressed in standard deviations, the range from -3 to +3 captures 99.7% of the theoretical normal distribution. All QQ plots approximate to normality at central values but diverge at the tails of the theoretical normal distribution.

| 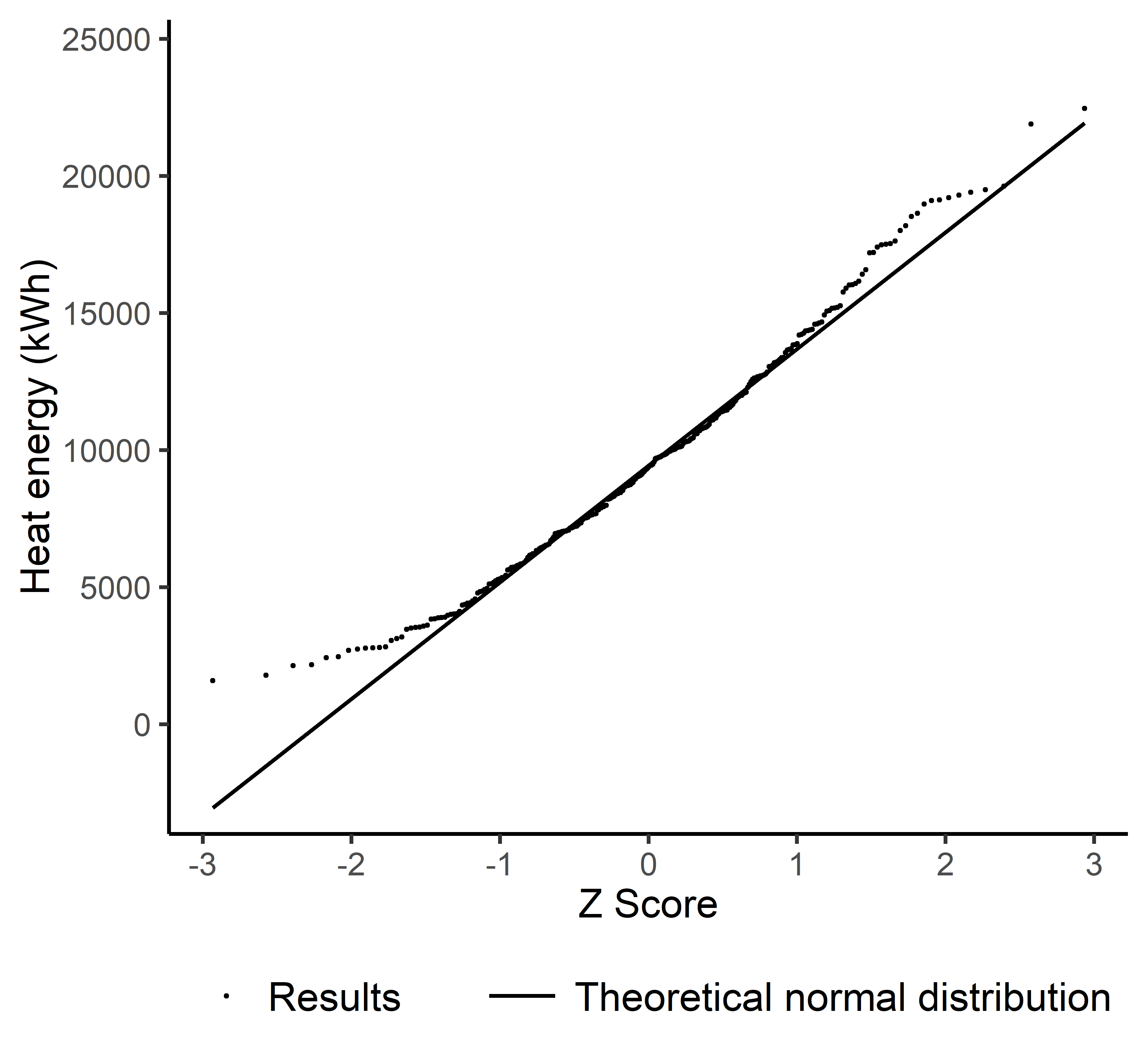  TH03 as-built | 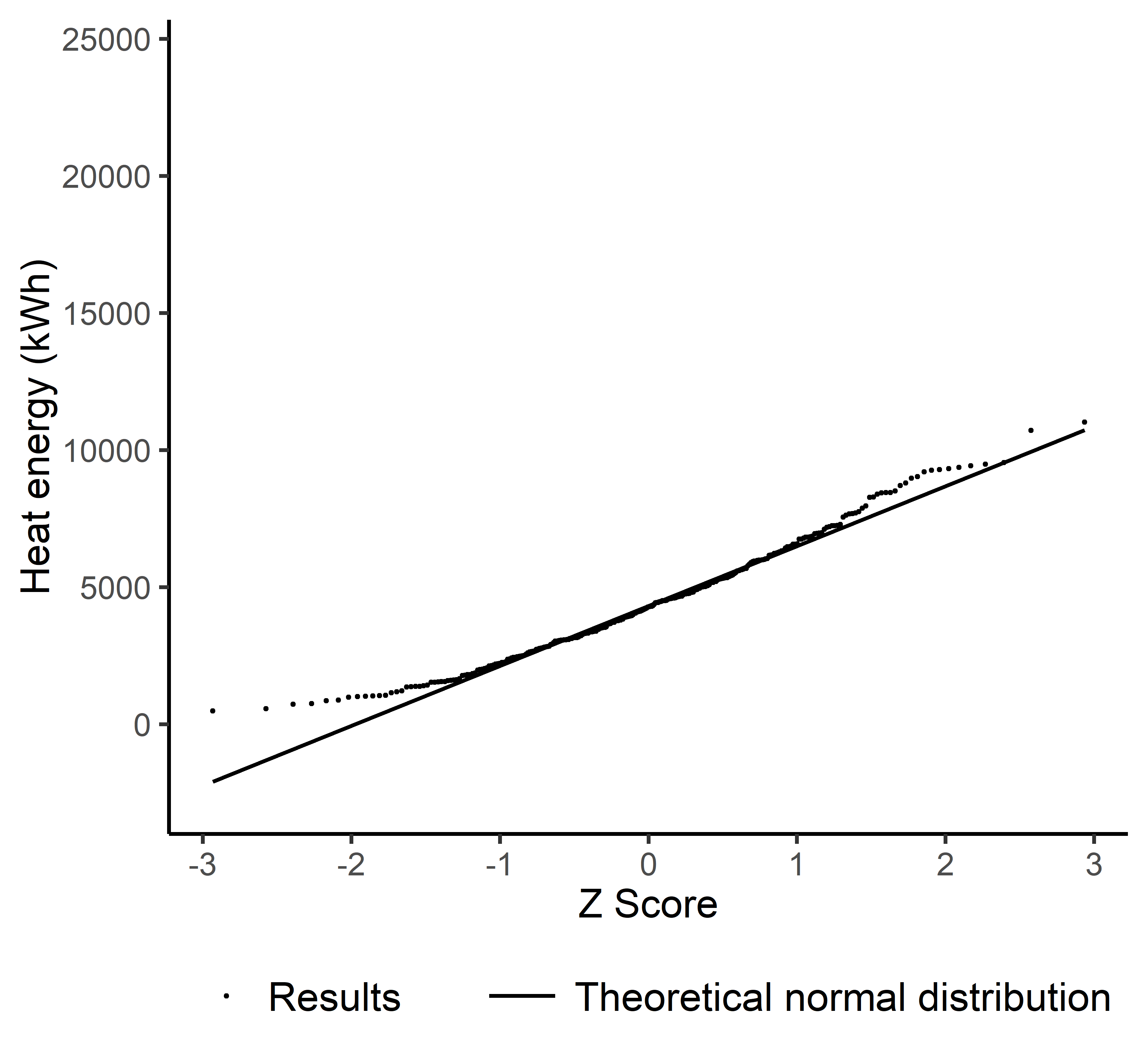  TH03 retrofit |
| --- | --- |
| 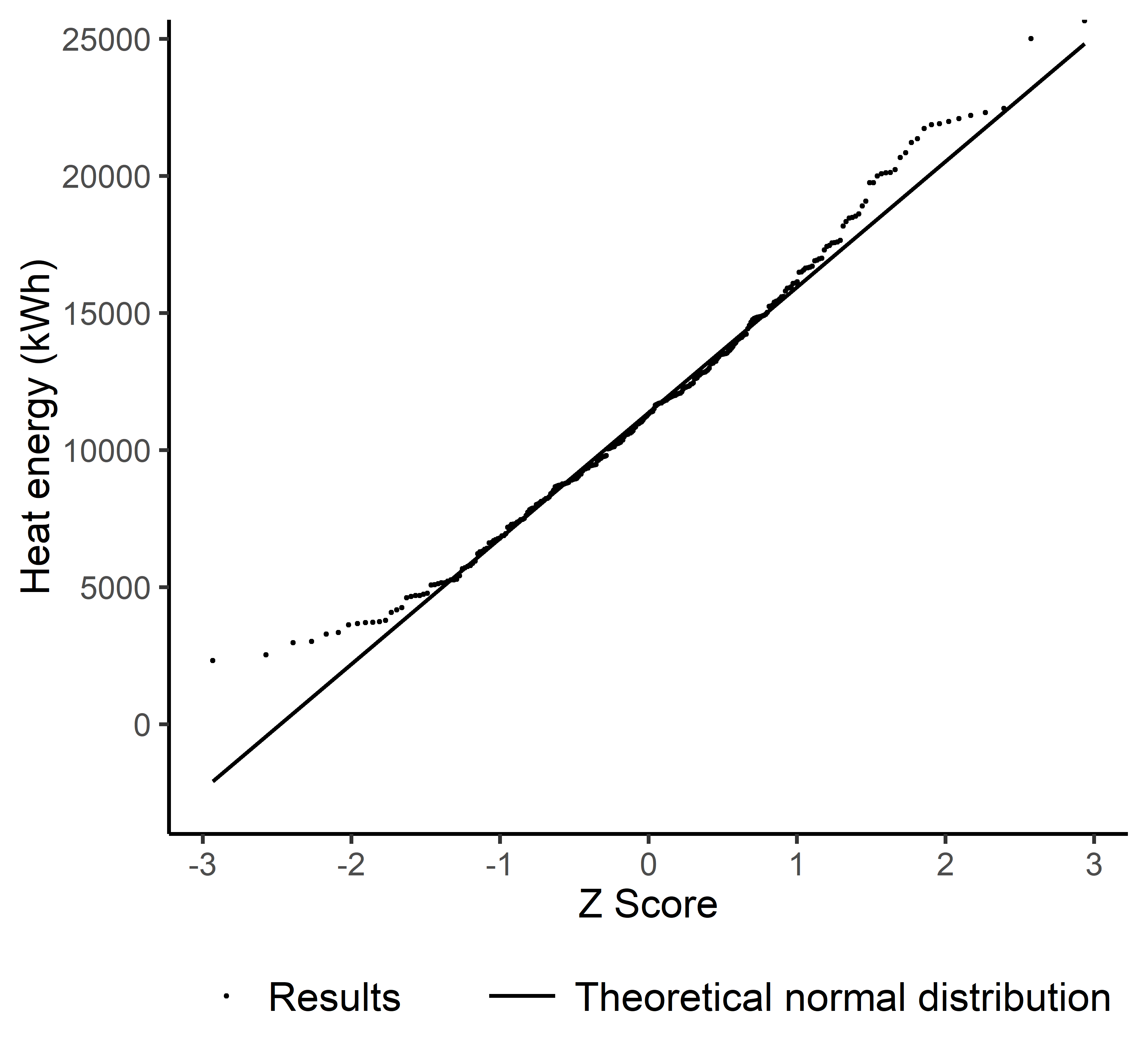  TH06 as-built | 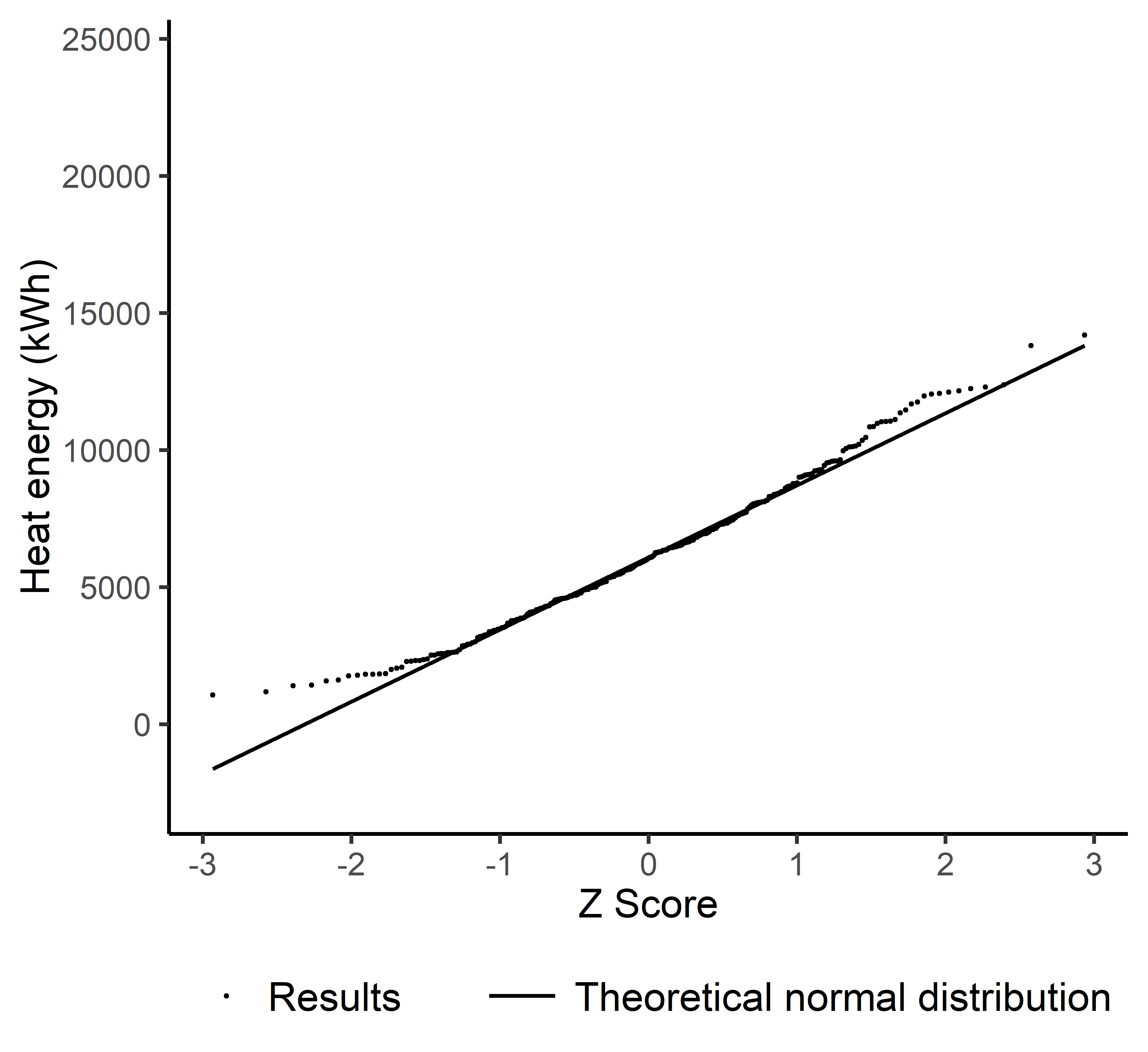  TH06 retrofit |
| 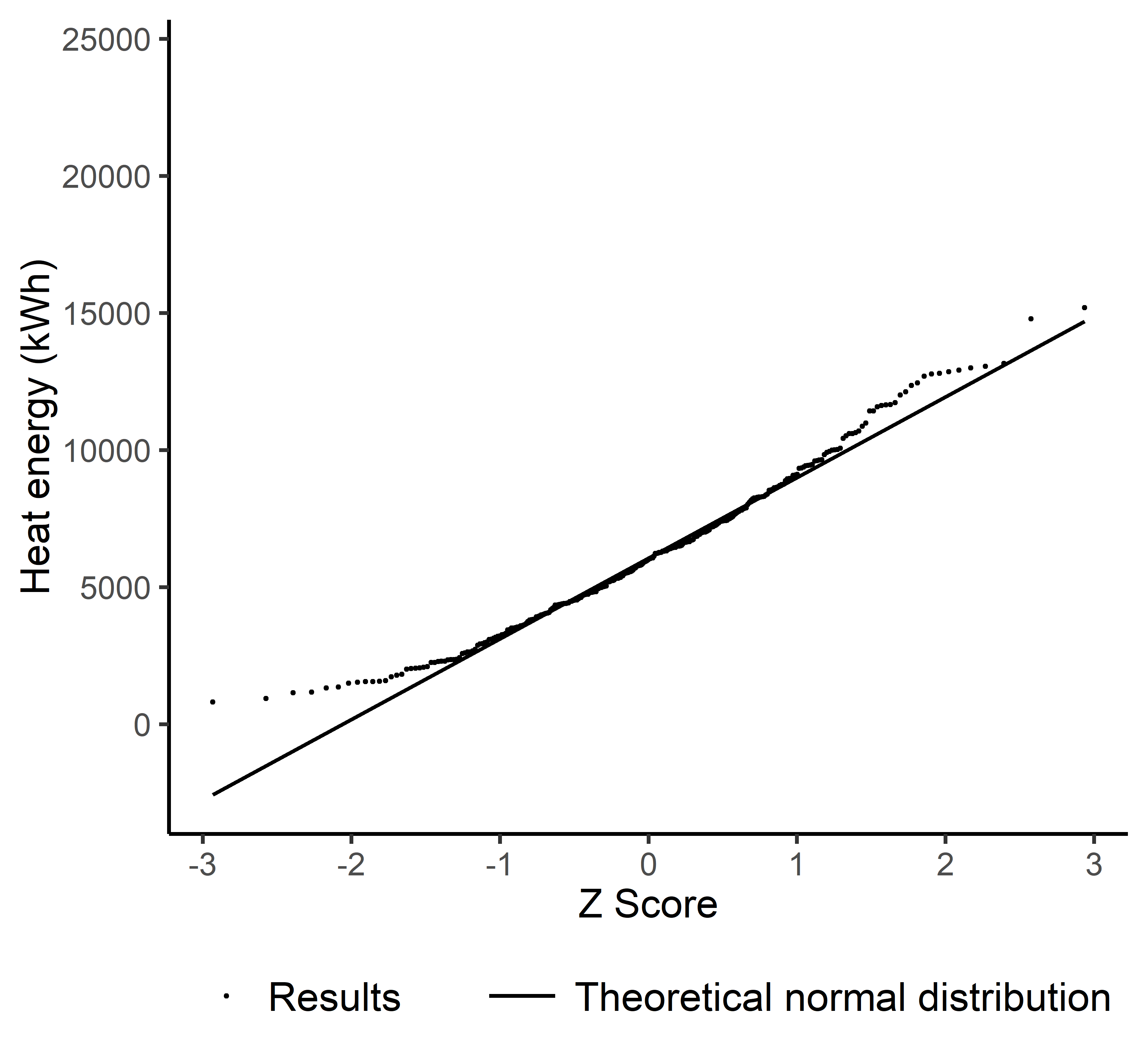  TH07 as-built | 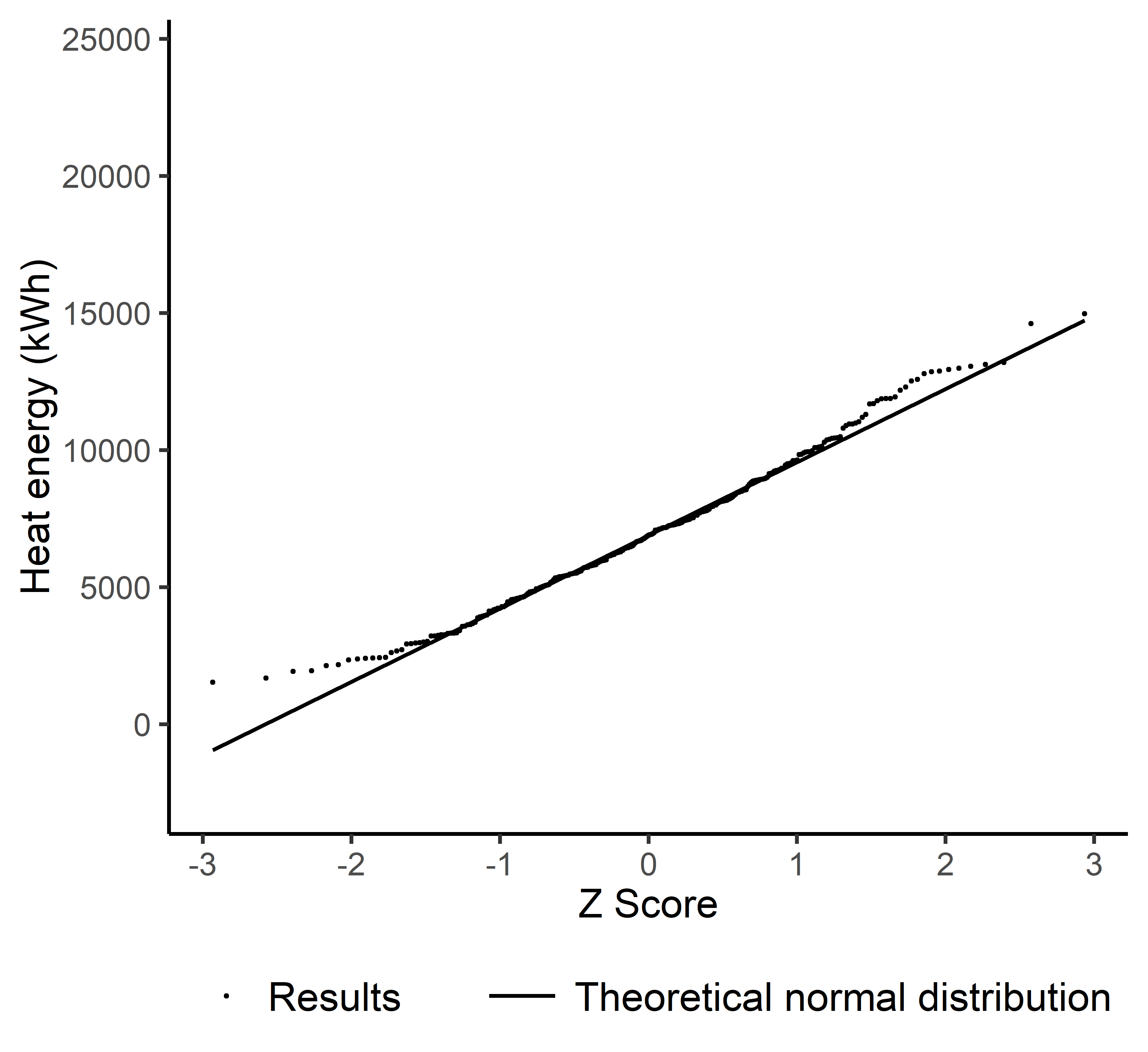 TH07 retrofit |

**Fig. S1** Quantile-quantile plots of yearly heat-energy usage by as-built and retrofit archetypes under the *constant* heating pattern. Each archetype simulated under the same two sets (n = 300) of as-built setpoints and retrofit setpoints.

| 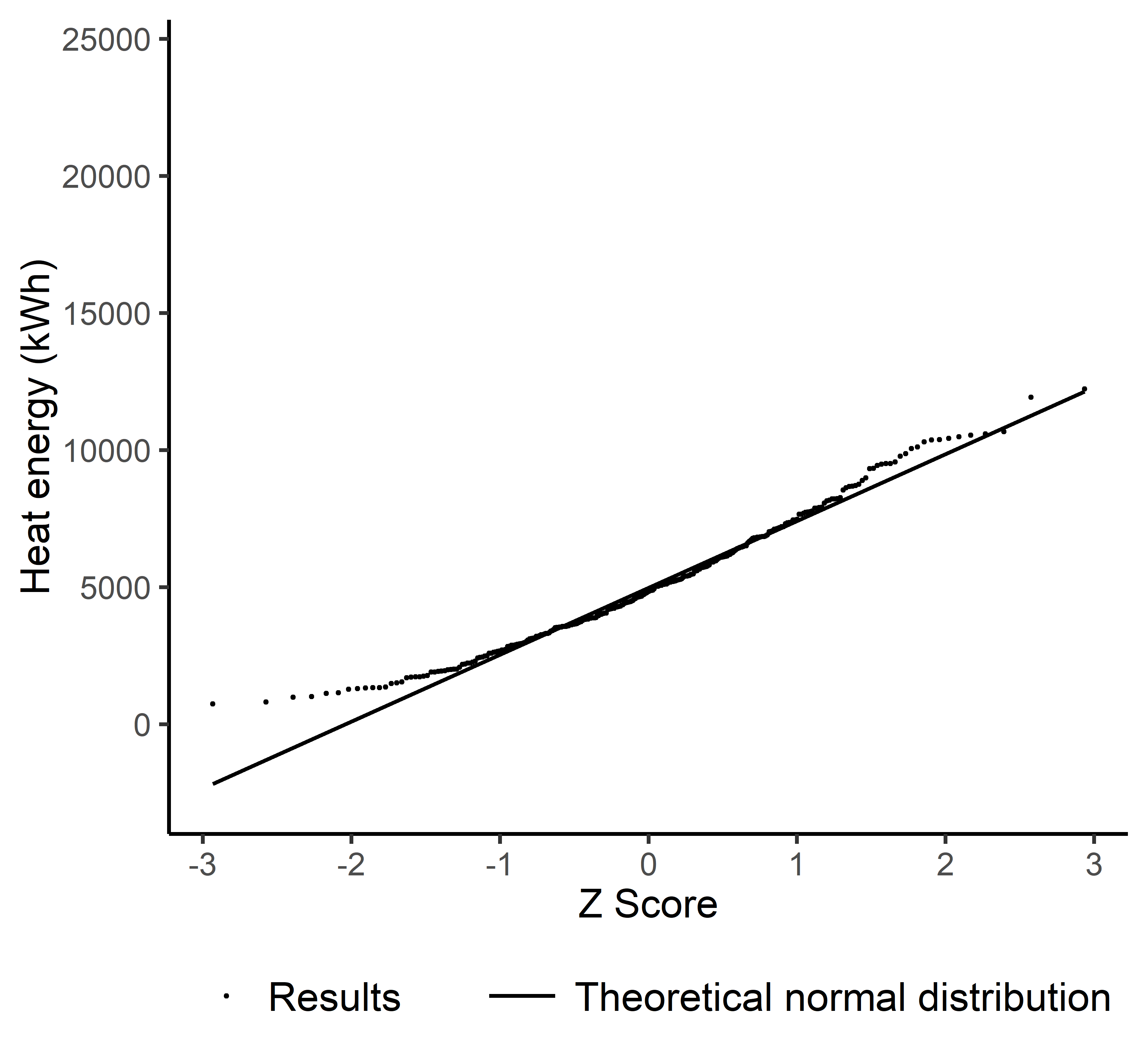  TH03 as-built | 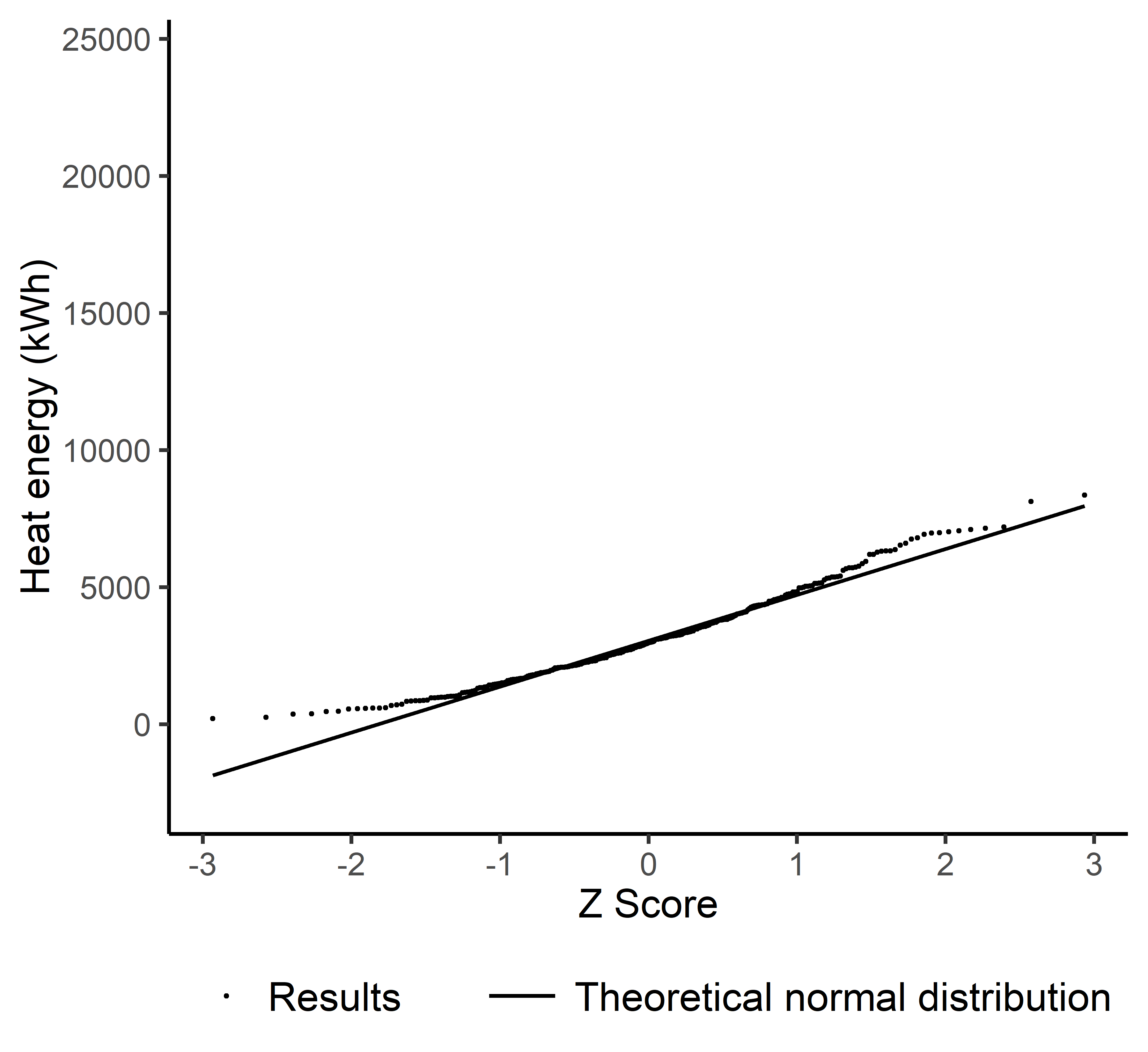  TH03 retrofit |
| --- | --- |
| 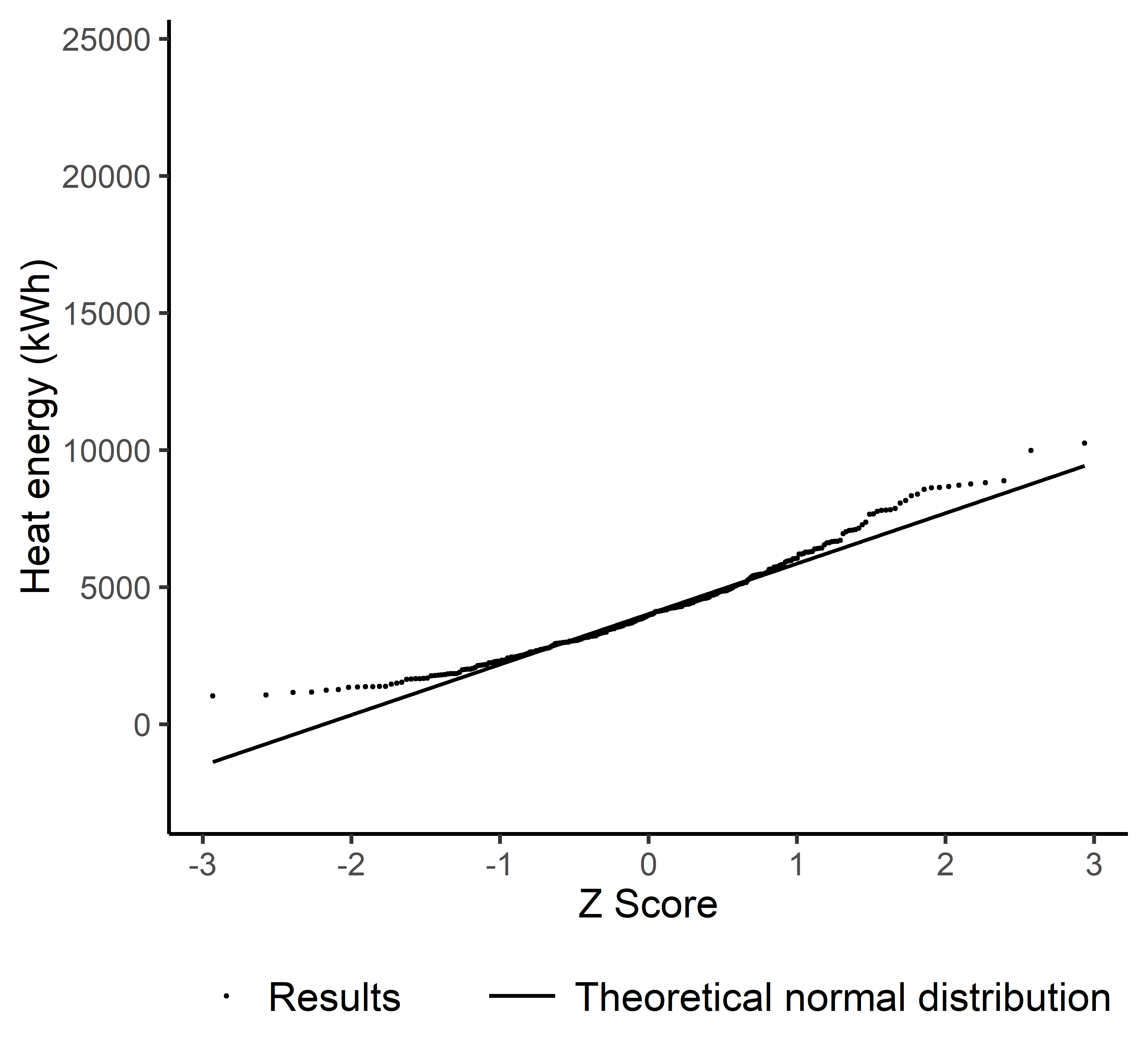  TH06 as-built | 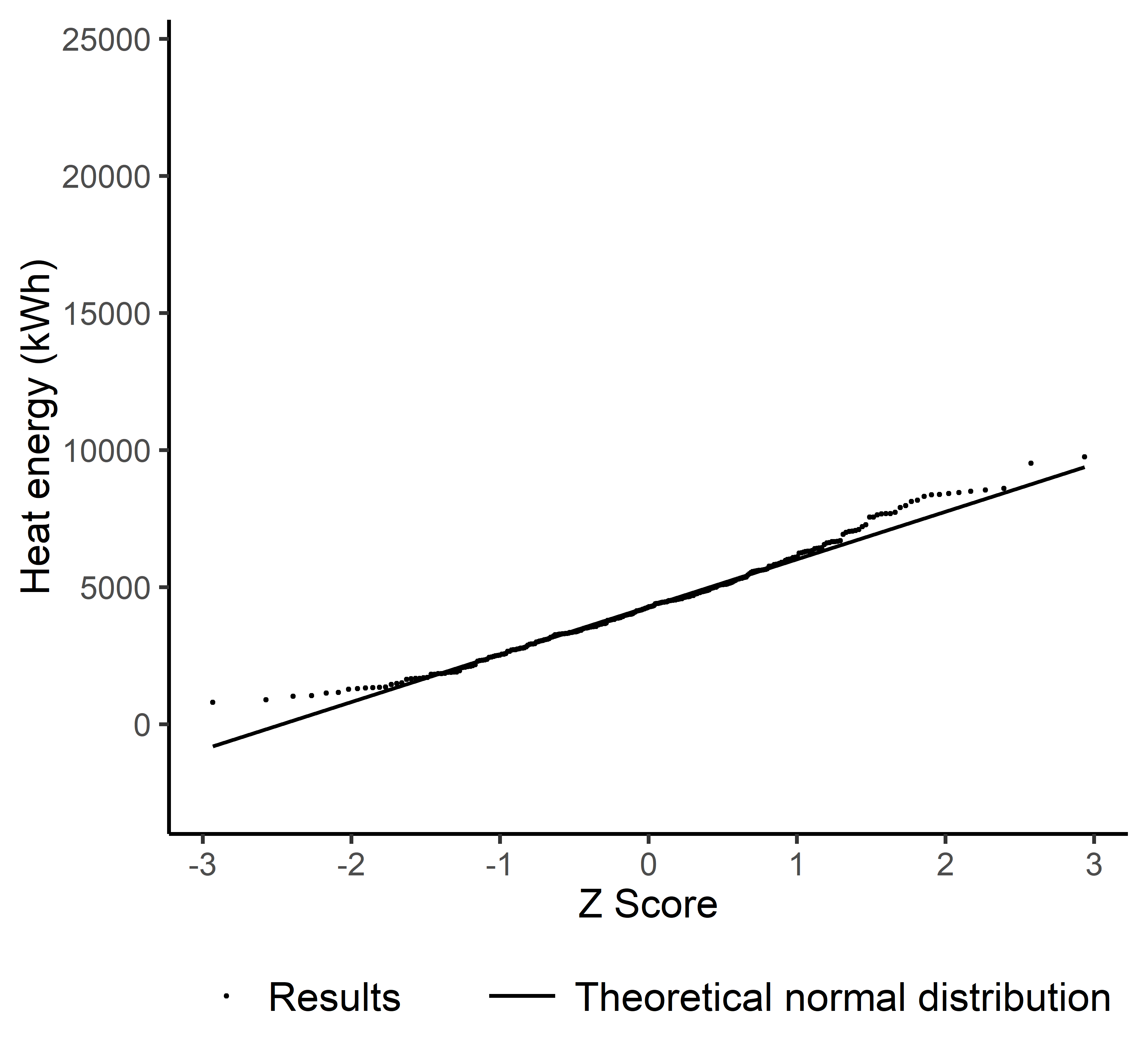  TH06 retrofit |
| 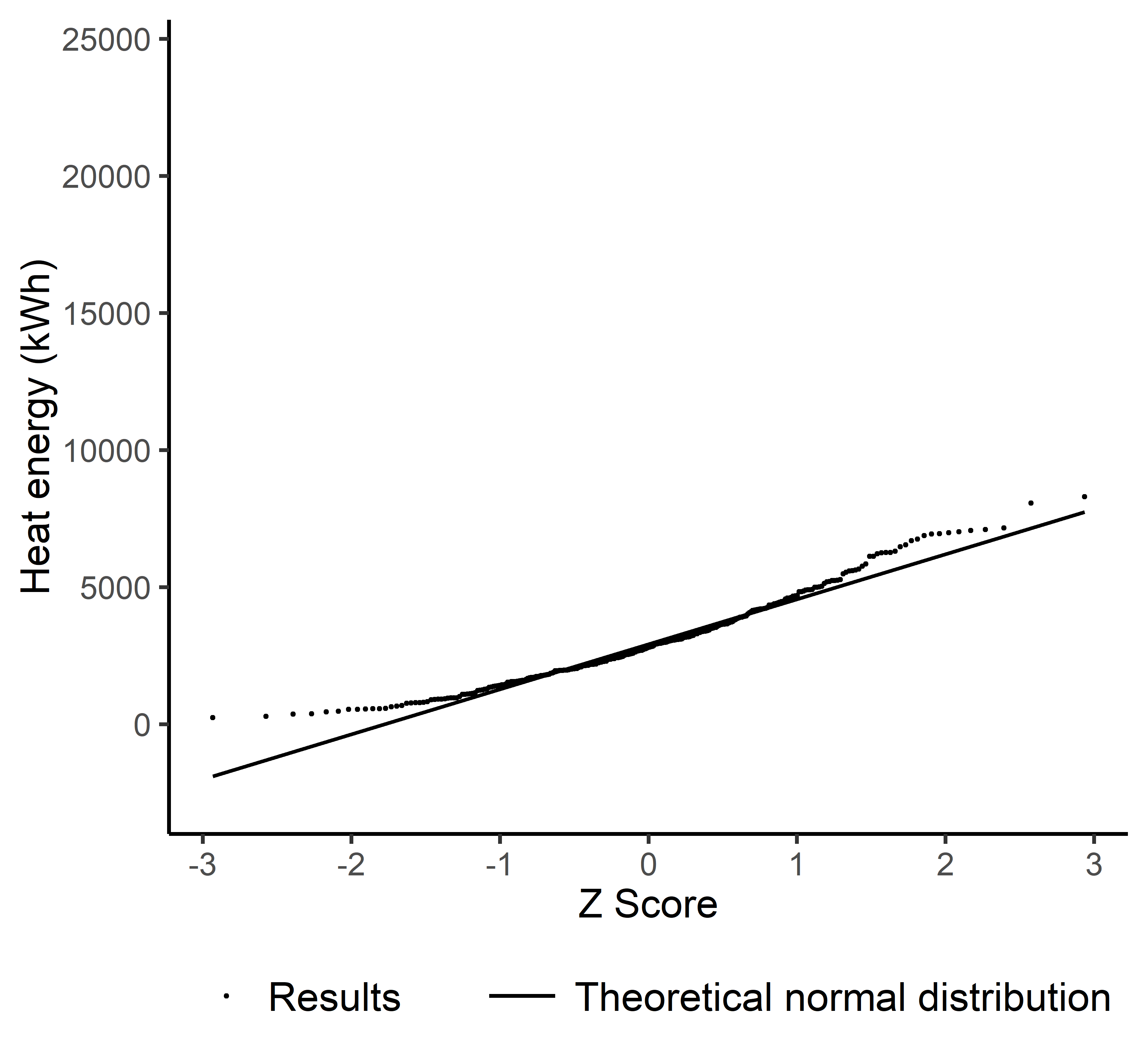  TH07 as-built | 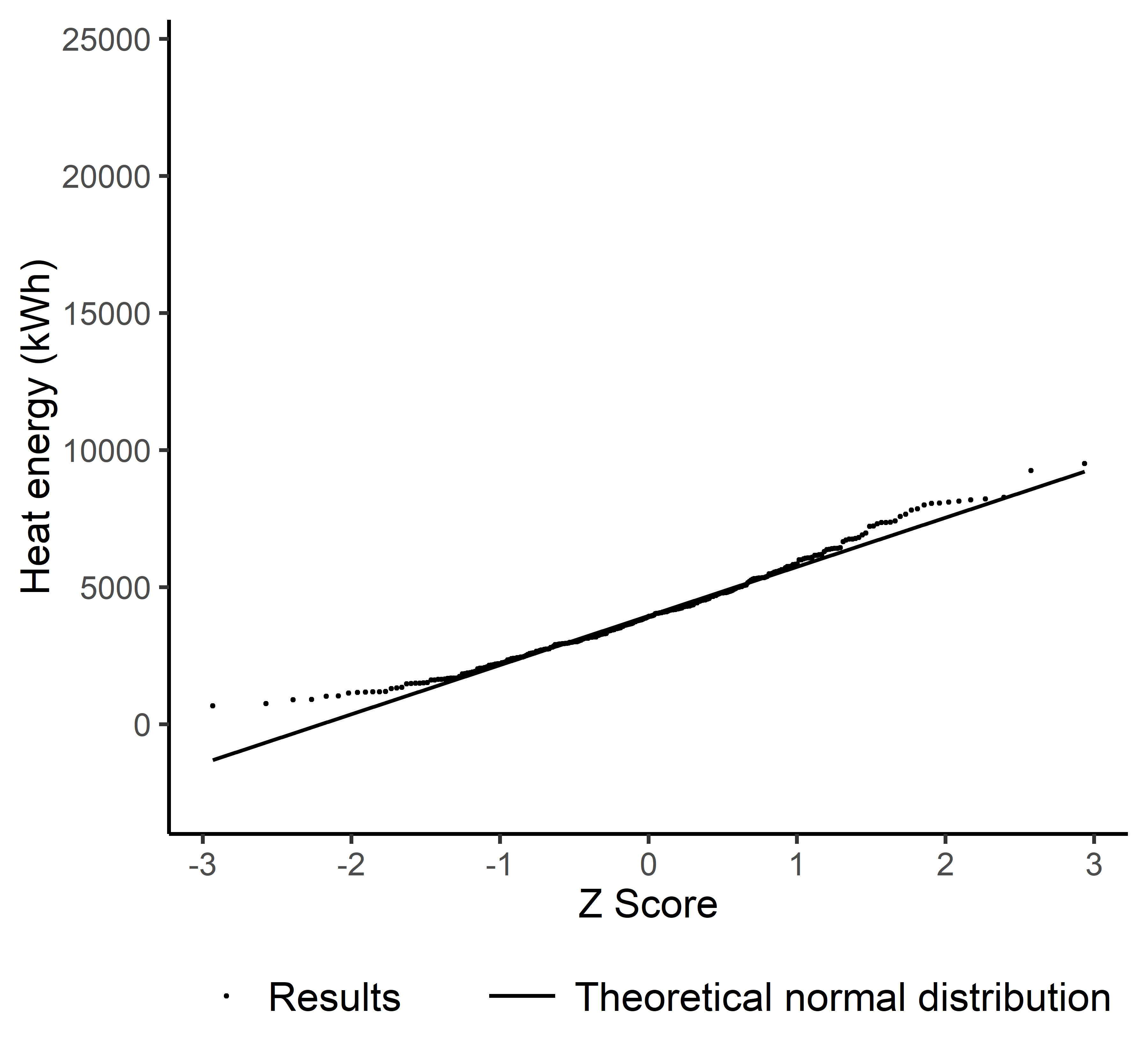 TH07 retrofit |

**Fig. S2** Quantile-quantile plots of yearly heat-energy usage by as-built and retrofit archetypes under the *single* heating pattern. Each archetype simulated under the same two sets (n = 300) of as-built setpoints and retrofit setpoints.

| 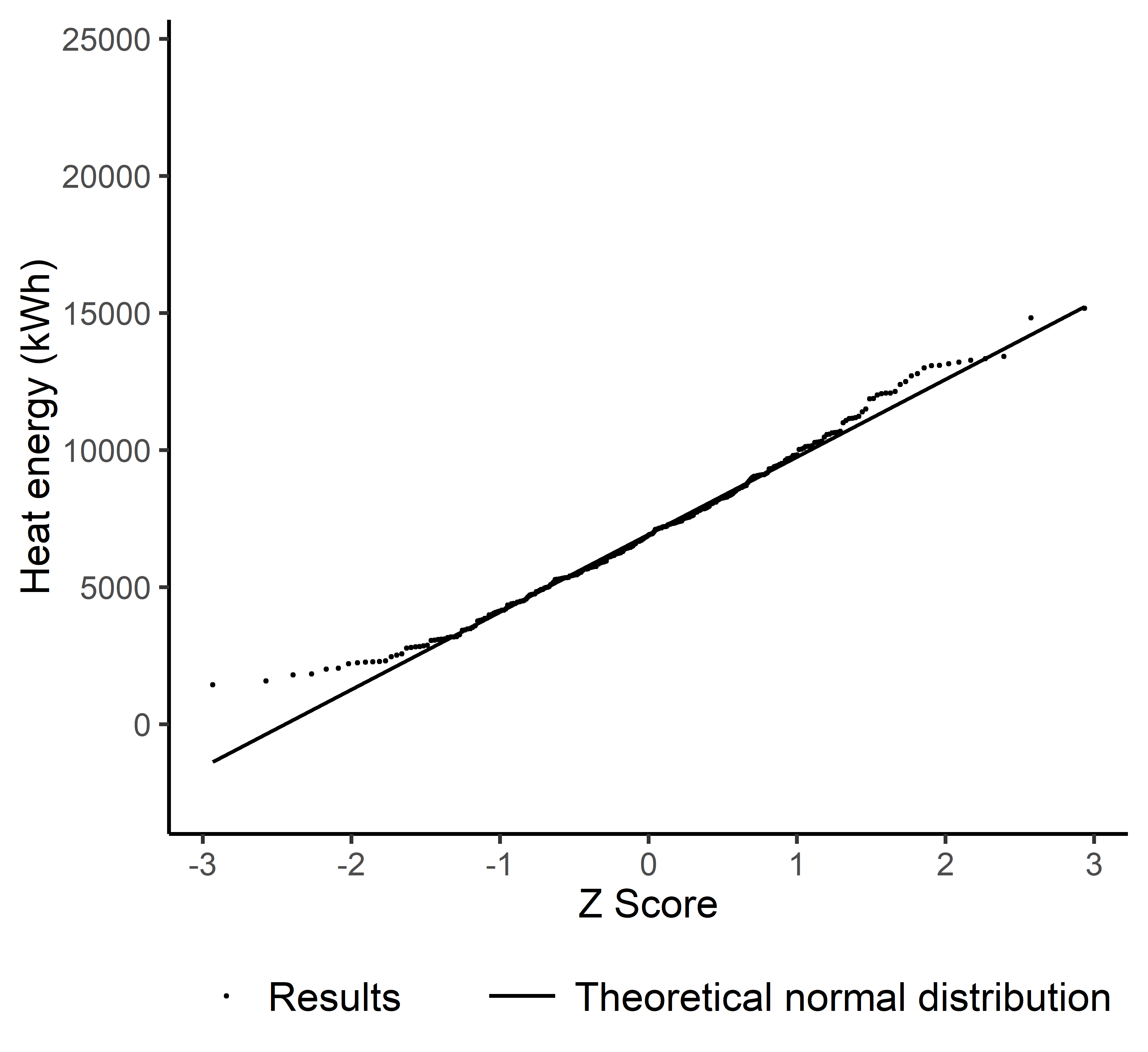  TH03 as-built | 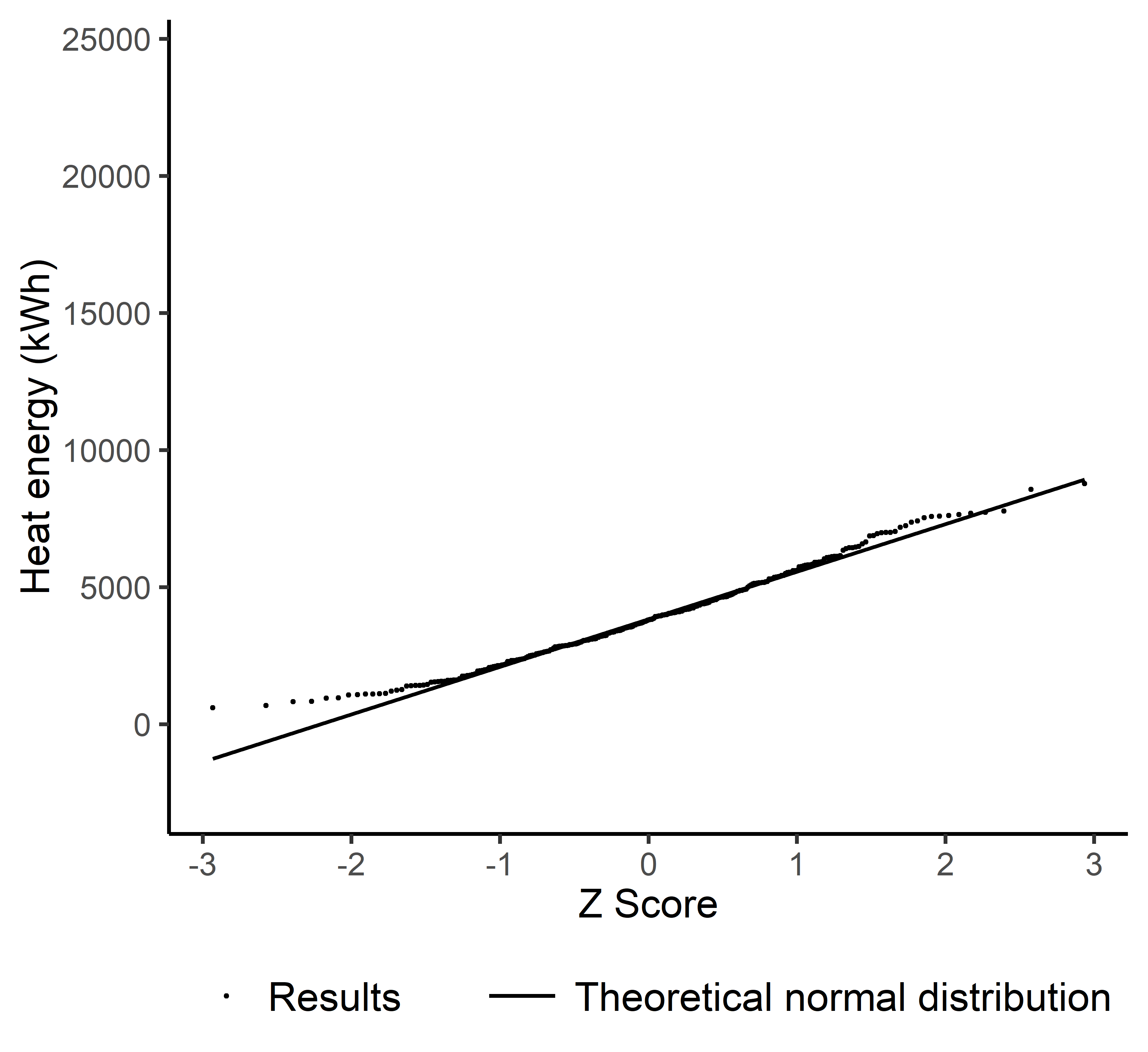  TH03 retrofit |
| --- | --- |
| 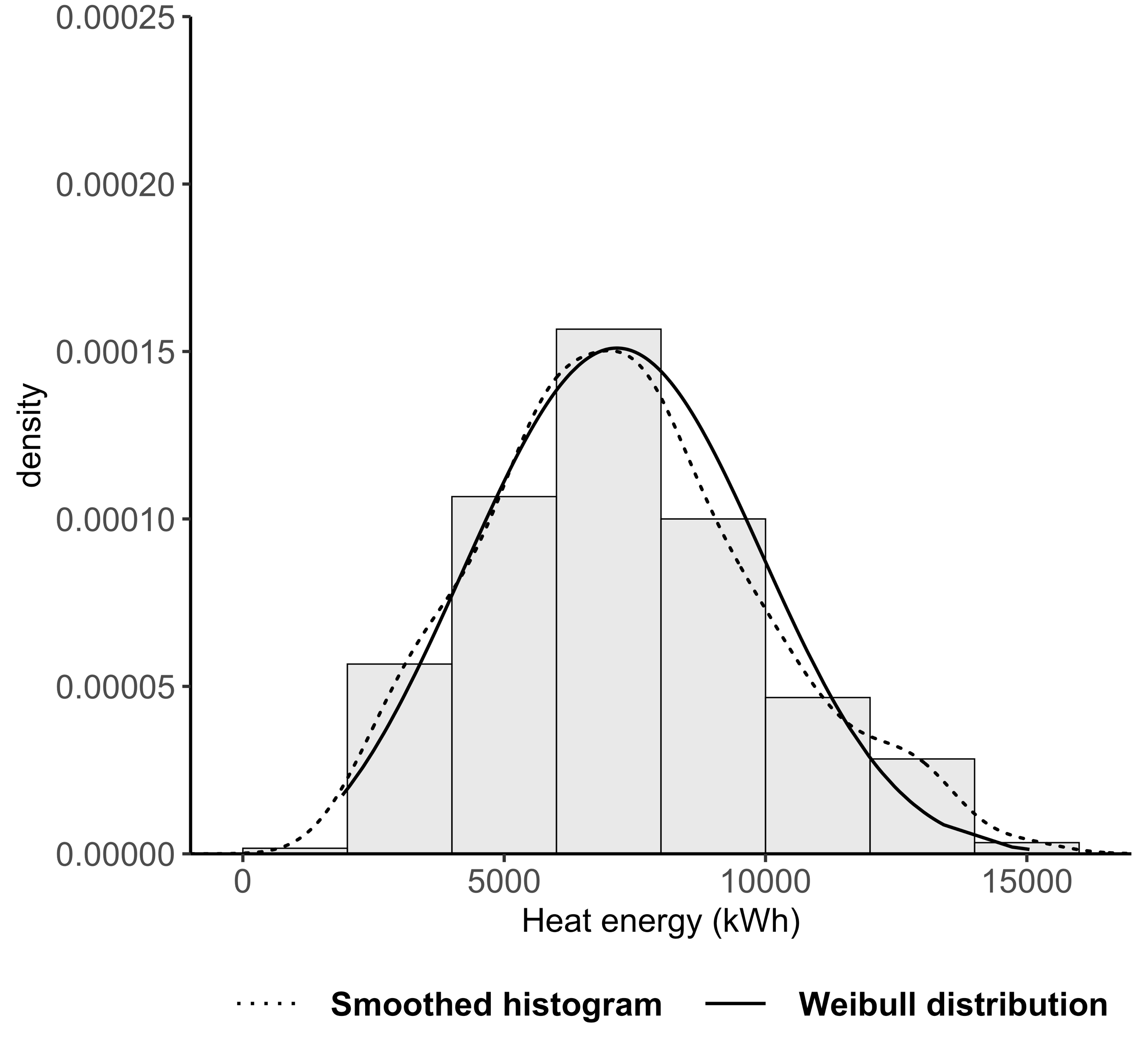  TH06 as-built | 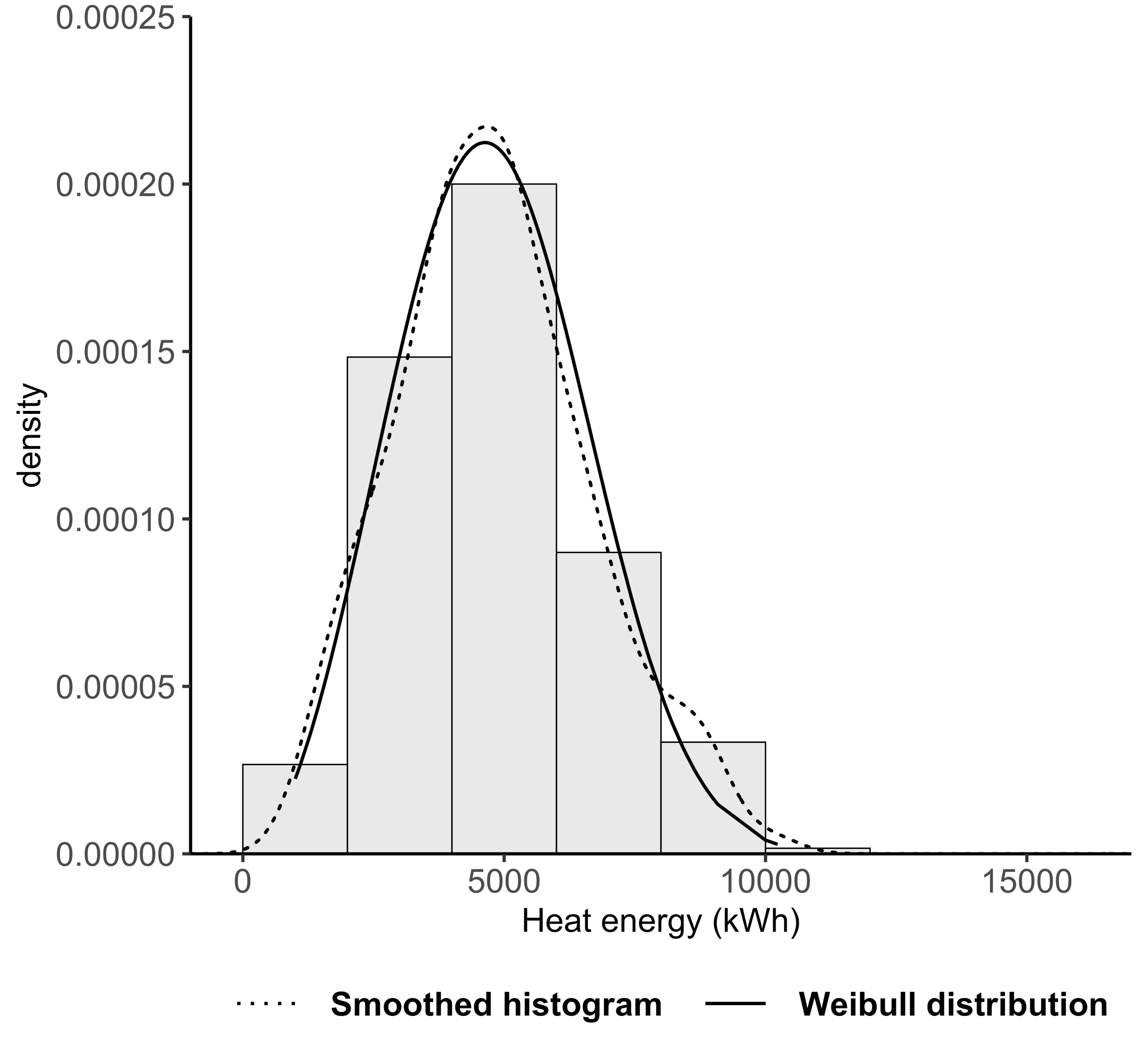  TH06 retrofit |
| 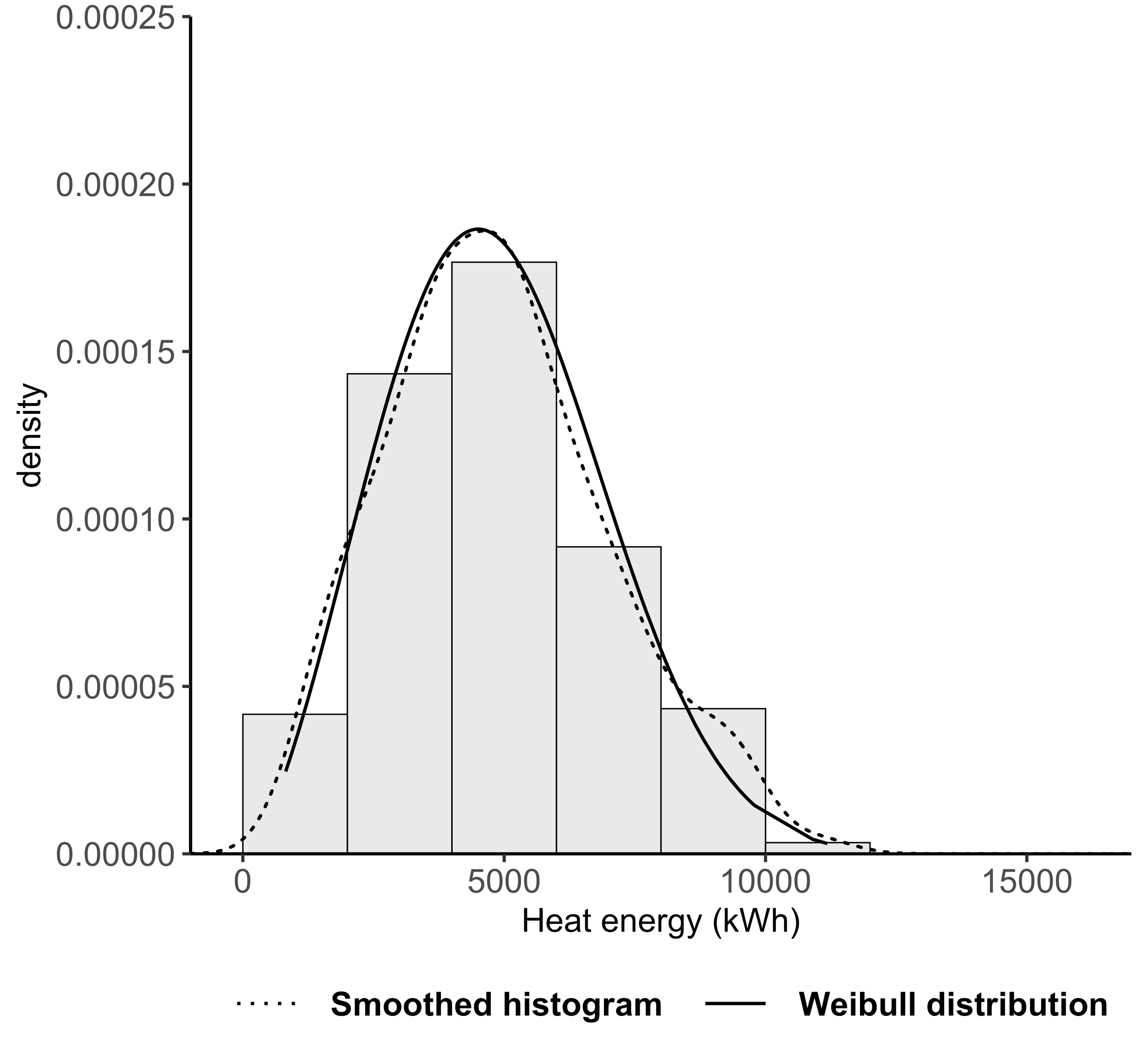  TH07 as-built | 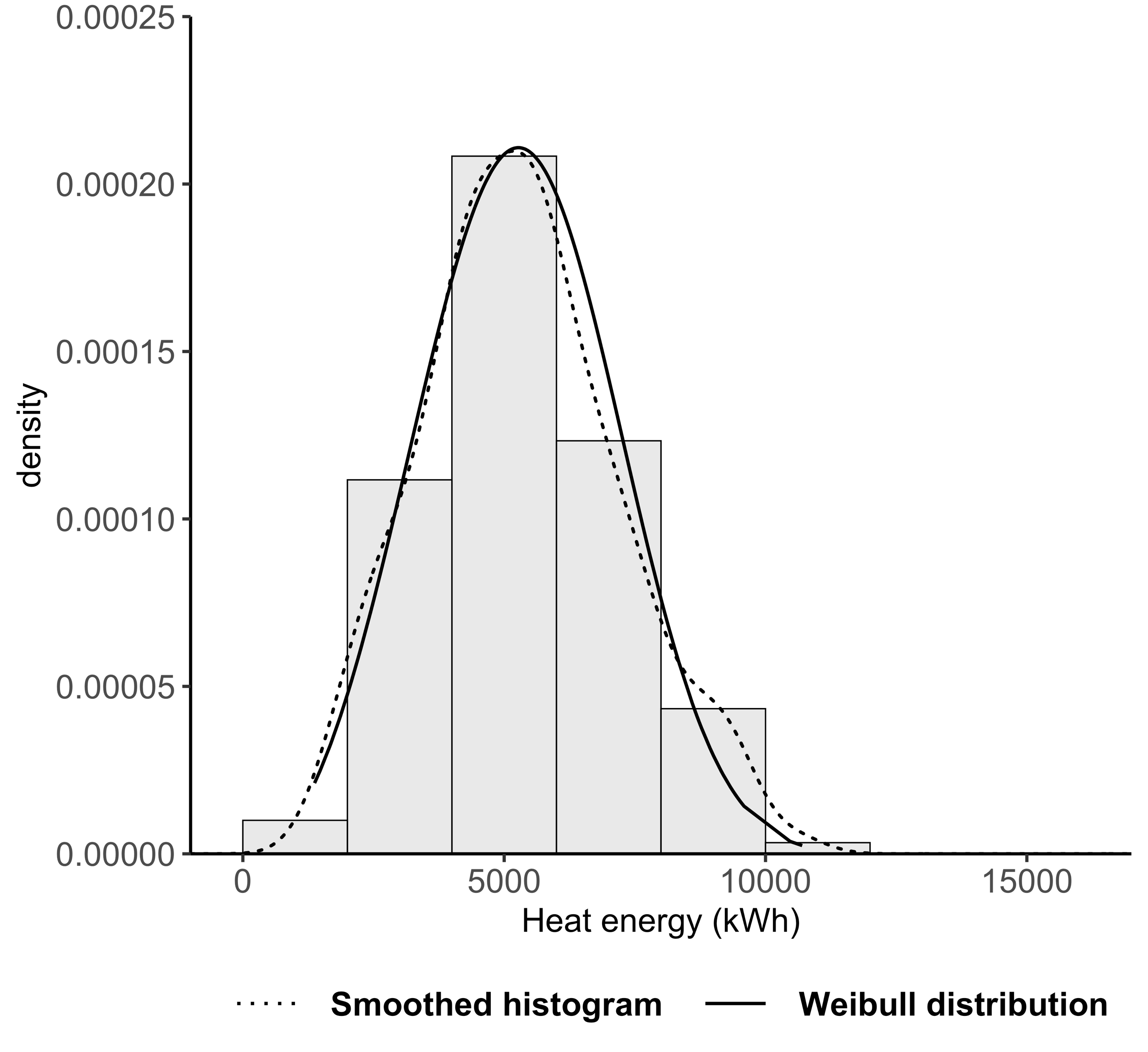 TH07 retrofit |

**Fig. S3** Quantile-quantile plots of yearly heat-energy usage by as-built and retrofit archetypes under the *double* heating pattern. Each archetype simulated under the same two sets (n = 300) of as-built setpoints and retrofit setpoints.
